# Granulocytes Impose a Tight Bottleneck upon the Gut Luminal Pathogen Population during *Salmonella* Typhimurium Colitis

**DOI:** 10.1371/journal.ppat.1004557

**Published:** 2014-12-18

**Authors:** Lisa Maier, Médéric Diard, Mikael E. Sellin, Elsa-Sarah Chouffane, Kerstin Trautwein-Weidner, Balamurugan Periaswamy, Emma Slack, Tamas Dolowschiak, Bärbel Stecher, Claude Loverdo, Roland R. Regoes, Wolf-Dietrich Hardt

**Affiliations:** 1 Eidgenössische Technische Hochschule Zürich, Institute of Microbiology, Zurich, Switzerland; 2 Max von Pettenkofer-Institut, München, Germany; German Center for Infection Research (DZIF), partner site Ludwig Maximilian University of Munich, Munich, Germany; 3 Eidgenössische Technische Hochschule Zürich, Institute of Integrative Biology, Zurich, Switzerland; Purdue University, United States of America

## Abstract

Topological, chemical and immunological barriers are thought to limit infection by enteropathogenic bacteria. However, in many cases these barriers and their consequences for the infection process remain incompletely understood. Here, we employed a mouse model for *Salmonella* colitis and a mixed inoculum approach to identify barriers limiting the gut luminal pathogen population. Mice were infected via the oral route with wild type *S.* Typhimurium (*S.* Tm) and/or mixtures of phenotypically identical but differentially tagged *S.* Tm strains (“WITS”, wild-type isogenic tagged strains), which can be individually tracked by quantitative real-time PCR. WITS dilution experiments identified a substantial loss in tag/genetic diversity within the gut luminal *S.* Tm population by days 2–4 post infection. The diversity-loss was not attributable to overgrowth by *S.* Tm mutants, but required inflammation, Gr-1^+^ cells (mainly neutrophilic granulocytes) and most likely NADPH-oxidase-mediated defense, but not iNOS. Mathematical modelling indicated that inflammation inflicts a bottleneck transiently restricting the gut luminal *S.* Tm population to approximately 6000 cells and plating experiments verified a transient, inflammation- and Gr-1^+^ cell-dependent dip in the gut luminal *S.* Tm population at day 2 post infection. We conclude that granulocytes, an important clinical hallmark of *S.* Tm-induced inflammation, impose a drastic bottleneck upon the pathogen population. This extends the current view of inflammation-fuelled gut-luminal *Salmonella* growth by establishing the host response in the intestinal lumen as a double-edged sword, fostering and diminishing colonization in a dynamic equilibrium. Our work identifies a potent immune defense against gut infection and reveals a potential Achilles' heel of the infection process which might be targeted for therapy.

## Author Summary

*Salmonella* Typhimurium can colonize the human intestine and cause severe diarrhea. In recent years, it has become clear that this pathogen profits from inflammatory changes in the intestinal lumen, as the inflamed gut helps *Salmonella* to out-compete the resident microbiota. Granulocytes transmigrating into the gut lumen were found to “foster” luminal *Salmonella* growth by providing nutrients (used by *Salmonella*, not the microbiota) and by releasing growth inhibitors affecting the microbiota, but not the pathogen. In this study, we extend this “fostering” concept by showing that gut luminal *Salmonella* Typhimurium population is itself surprisingly vulnerable to the host's inflammatory response. Indeed, inflammation reduces the size of the gut luminal *Salmonella* population by as much as 10^5^-fold at day 2 post infection. Thus, triggering of mucosal inflammation is in fact a double-edged sword by providing *S.* Typhimurium with a relative growth advantage against the microbiota in the gut lumen and by killing 99.999% of the gut luminal pathogen population at day 2. However, the pathogen population can recover and grow up again during the subsequent days. This changes the current view: Inflammation is not simply “beneficial” for the pathogen in the gut lumen. Instead, pathogen growth in the inflamed gut must be considered as an equilibrium between inflammation-inflicted killing and fostering growth of the surviving bacteria.

## Introduction

Acute infections constitute highly complex interactions between pathogens and their hosts. The complexity arises from dynamic changes in pathogen gene expression, pathogen growth, barriers limiting the initial colonization and host defenses which limit further pathogen growth and survival during the course of an infection. Identifying the relevant interactions and how they affect the progression of the disease is of great value for understanding the infection process and may reveal new targets for prevention or therapy.

Mixed inoculation provides a powerful approach to decipher pathogen-host interactions [1]. In such experiments, genetic markers carried by some members of the pathogen population are used to follow how the pathogen population disseminates, grows or is killed during the course of an infection [2]–[13]. This can reveal “barriers”, which limit the infection. Barriers can be of varying nature, including chemical barriers (i.e. antimicrobial peptides, stomach acid), physical obstacles (e.g. the mucus layer separating gut luminal bacteria from the epithelial surface [14]) or immune responses killing the pathogen. Such barriers can impose “bottlenecks” onto the pathogen population which can be detected as loss of marker diversity. Thus, barriers are important characteristics of an infection process as they indicate how hosts can interfere with pathogen colonization and survival.

We employed a mixed inoculum approach to study *Salmonella enterica* subspecies 1 serovar Typhimurium (termed *S.* Tm hereafter) growth in the inflamed gut using the well-established streptomycin mouse model for *Salmonella* colitis [15]. In this model, the resident microbiota is transiently suppressed by a single dose of streptomycin [16]. This bypasses the initial phase of the natural infection where the pathogen has to competitively grow in the face of an intact, dense microbiota [17] and allows us to focus on the next stage where the pathogen triggers disease and grows in the inflamed gut [15], which is still incompletely understood.

*S.* Tm mainly elicits mucosal inflammation via virulence factors encoded in genomic islands, i.e. the SPI-1 and SPI-2 type III secretion systems [18], [19]. The inflammatory response has been shown to foster efficient colonization of the host's gut lumen by the pathogen, as the milieu in the inflamed gut can help the pathogen to outcompete and/or suppress the resident microbiota [20]. Some of the molecular mechanisms have been identified [15]. This includes the elicitation of antimicrobial peptides which kill some of the microbiota (but not *S.* Tm; [21]), the limitation of iron and zinc uptake by microbiota species (but not *S.* Tm; [22], [23]) and the provision of terminal electron acceptors fuelling *S.* Tm growth by anaerobic respiration [24], [25]. These findings have established *S.* Tm as a pathogen subverting gut luminal inflammation to efficiently colonize this niche. However, it was previously unclear whether the inflammatory response also inflicts an additional barrier limiting pathogen colonization of the gut. This would seem reasonable as inflammation is generally mounted to fight infection, i.e. in infected host tissues [26], [27]. Indeed, some studies have observed transient, 10-fold reductions of gut luminal pathogen loads at day 2 p.i. [20] and Reg3β, an antimicrobial peptide released by the inflamed mucosa, was found to kill fast-growing *S.* Tm cells [28].

In this study, we have employed a mixed inoculum approach to identify barriers limiting gut luminal colonization by *S.* Tm. Using *S.* Tm strains chromosomally tagged with bar coded sequences (WITS, wild-type isogenic tagged strains; [5], [6], [29]) we identified a pronounced bottleneck in the gut luminal pathogen population and oligo-clonal expansion post crisis. The barrier was attributable to pathogen-induced inflammation, in particular granulocytes, a prominent phagocytic cell type infiltrating the infected mucosa and the gut lumen. Our data extend the current paradigm by establishing that not only the tissue-infiltrating bacteria, but also the gut luminal pathogen population, can be temporarily restricted by the host's inflammatory response.

## Results

### WITS infections reveal neutral genetic diversity loss by *S.* Tm in the cecum lumen and feces of streptomycin pretreated mice

To identify barriers limiting gut luminal pathogen colonization, we monitored the *S*. Tm community composition by using seven wild-type isogenic tagged *S.* Tm strains (WITS), which are phenotypically identical (S1 Figure). Each WITS harbors a 40 nucleotide “bar code” between two pseudogenes which allow quantification of their relative proportion by rtqPCR [5], [6], [30]. In case of perturbations, the equal proportions of the WITS-tagged subpopulations will shift towards an uneven population structure in which some WITS are dominant while others disappear (i.e. loss of diversity; reduced “evenness”). Dilution of the tagged strains by untagged *S.* Tm^WT^ probes the degree of diversity loss (S1C Figure).

**Figure 1:**
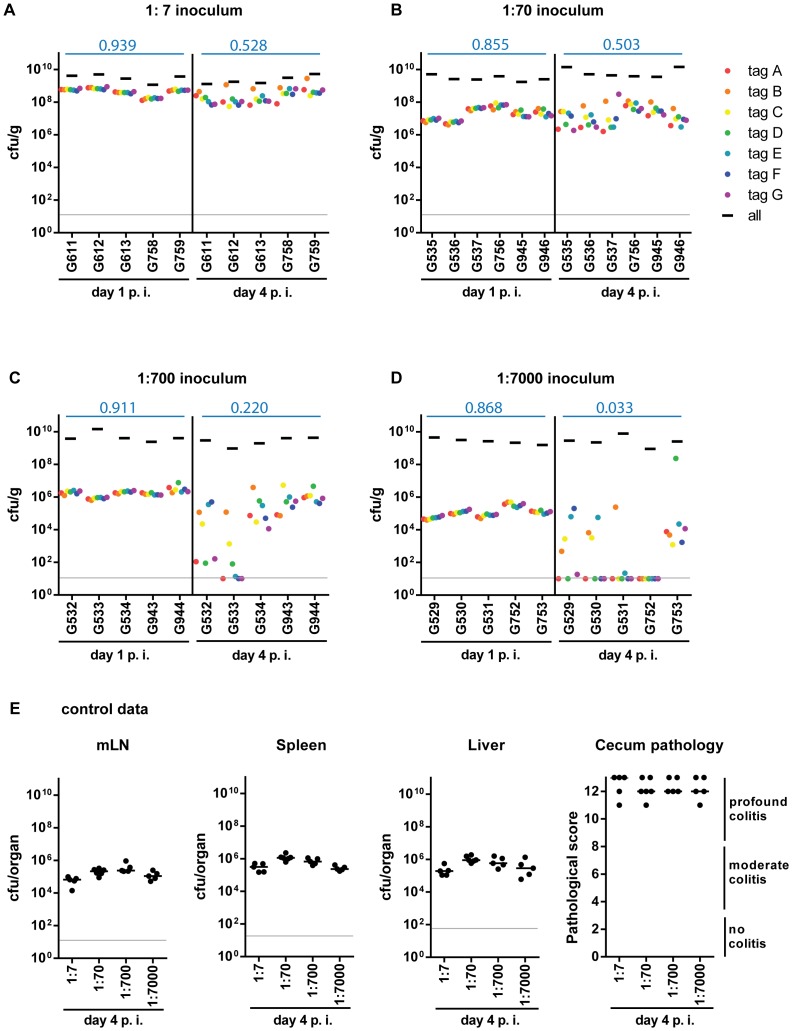
Systematic dilution experiments of the tagged *S.* Tm^WT^ strains allow assessing the level of WITS diversity loss. Infections were performed by diluting the tagged *S.* Tm^WT^ WITS with the untagged isogenic strain to a final proportion of (A) 1∶7, (B) 1∶70, (C) 1∶700, (D) 1∶7000 of each strain. The population composition was monitored in fecal samples at day 1 post infection (day 1 p.i.) and in cecal content at day 4 post infection (day 4 p.i.) of each mouse (individual mouse identifiers were plotted on the x-axis). The cfu/g fecal or cecal content for the whole population and each single tagged strain were plotted. Systemic spread (cfu per organ in mLN, spleen and liver) and cecal pathology on day 4 p.i. (E) confirmed a typical *S.* Tm^WT^-induced course of disease. Grey lines depict the detection limit for plating. Blue numbers indicate the median of the evenness indices of single mice.

First, we screened for optimal assay conditions by titrating the fraction of tagged WITS strains in the inoculum. A “1∶7” inoculum was prepared by mixing seven different WITS at an equal ratio (1∶1∶1∶1∶1∶1∶1; S1 Figure). The “1∶70”, 1∶700” and “1∶7000” inocula were generated by diluting the original WITS mixture with an untagged, isogenic wild type strain (1∶10, 1∶100 and 1∶1000, respectively). Streptomycin pretreated C57BL/6 mice were infected with the indicated inocula (5×10^7^ cfu total, by gavage; without sodium bicarbonate-mediated neutralization of stomach acid, 2 independent experiments using a total of 5 or 6 mice per group) and we monitored pathogen loads in the stool by plating fecal pellets (day 1 p.i.), the cecal content, the mesenteric lymph nodes (mLN), the livers and the spleens (day 4 p.i.). All animals featured high pathogen loads in the feces at day 1 and in the cecum lumen at day 4 p.i. (≈10^9^ cfu/g; Fig. 1A–D, black bars). Furthermore, we observed efficient colonization of the mLN, spleen and liver and all mice displayed profound cecum inflammation by day 4 p.i. (Fig. 1E). This is well in line with previous work [16], [19] and verified that the tags do not interfere with the infection process.

To detect diversity loss and identify optimal assay conditions, we quantified each WITS in the inoculum, the feces (day 1 p.i.) and the cecum content (day 4 p.i.). To analyze the WITS-diversity in a quantitative fashion, we have used the “evenness index”, an indicator commonly used to describe differences in the distribution of goods or money in a society ( = Gini-index [31]; see also S1 Figure). In our experiments, this index had a value close to 1 in the inoculum (0.913, median). Slight deviations (i.e. values from 0.9 to 1) are most likely attributable to technical errors (e.g. pipetting errors, PCR bias, cfu determination, DNA recovery, enrichment culture, etc.), not loss of genetic diversity in the biological system (S2 Figure).

At day 1 p.i., all WITS were detectable in the feces, no matter which inoculum was applied, and evenness indices ranged from 0.914–0.950 (1∶7 inoculum; median = 0.939) to 0.833–0.893 (1∶7000 inoculum; median = 0.868; Fig. 1A–D). This high degree of evenness indicated that the population did not encounter detectable bottlenecks, “selective sweeps” [32] or other effects limiting the genetic diversity during the transit through the gastrointestinal tract or the initial colonization of the host's large intestinal lumen by day 1 p.i.

In stark contrast, we detected dramatically reduced evenness indices of cecum luminal *S.* Tm populations at day 4 p.i., in particular at the highest WITS-dilutions (1∶7000; median = 0.033; Table 1). In the 5 mice, 18 of the 35 WITS were lost from the cecum lumen at day 4, and one animal did not retain any detectable WITS (mouse #G752; Fig. 1D). The identity of the dominant WITS and the WITS lost from the population differed from animal to animal and from experiment to experiment (Fig. 1A–D, see color code). These observations provided additional evidence that all WITS had equivalent fitness in our model and suggested that the diversity is reduced by a stochastic process.

**Table 1:** Statistics on population evenness.

Statistics for different dilutions (Fig. 1&2) at day 4 p.i., cecum		
Mann-Whitney U test	p-value	Summary
1∶7 vs. 1∶70	0.7706	ns
1∶7 vs. 1∶700	0.0317	*
1∶7 vs. 1∶7000	0.0016	**
1∶70 vs. 1∶700	0.0823	ns
1∶70 vs. 1∶7000	0.0013	**
1∶700 vs. 1∶7000	0.0287	*

Statistics for population evenness in fecal vs. cecal content (Fig. 2)		
Mann-Whitney U test	p-value	Summary
day 1 p.i.	0.4127	ns
day 2 p.i.	0.5238	ns
day 3 p.i.	0.9444	ns
day 4 p.i.	0.9714	ns

Statistics for different strain backgrounds (Fig. 3&4) at day 4 p.i., cecum		
Mann-Whitney U test	p-value	Summary
WT vs. SPI1&2	0.0002	***
WT vs. SPI1	0.0016	**
WT vs. SPI2	0.0003	***
WT vs. SPI2 in WT	0.4119	ns
SPI1&2 vs. SPI1	0.2222	ns
SPI1&2 vs. SPI2	0.0541	ns
SPI1 vs. SPI2	0.0732	ns
SPI2 vs. SPI2 in WT	0.0023	**

Statistics for Gr-1 depletion (Fig. 6), day 4 p.i., cecum		
Mann-Whitney U test	p-value	Summary
PBS vs. Gr-1 depletion	0.0173	*
129 Sv/Ev vs. C57BL/6 (1∶7000)	0.1066	ns

Formally, the reduced evenness observed at higher WITS-dilutions could be explained either by bottlenecks in the pathogen population or by selective sweeps (e.g. up-growth of fitter *S.* Tm mutants [33]). The latter was ruled out in competitive infections with re-isolated clones. These control experiments verified that (in general), the dominant WITS-isolates retained the fitness of the original strain (S3 Figure). Thus, selective sweeps [32] of beneficial mutations cannot explain the loss of WITS-diversity in the experiment shown in Fig. 1. Therefore, these data provided a first hint that a bottleneck might limit the diversity of the gut luminal pathogen population between days 1–4 post infection.

Taken together, our data suggested that a population-bottleneck explains the reduced evenness index at day 4 p.i. Equivalent observations were made in an *Nramp*-positive mouse line (129SvEv; see below), indicating that the barrier reducing the evenness may be a general feature, independent of the mouse line used. The gut luminal *S.* Tm population should encounter this bottleneck after day one and before day 4 p.i. The 1∶7000 inoculum seemed well suited for further analysis of this phenotype.

### The gut luminal pathogen population encounters a pronounced bottleneck at day 2 p.i

**Figure 2:**
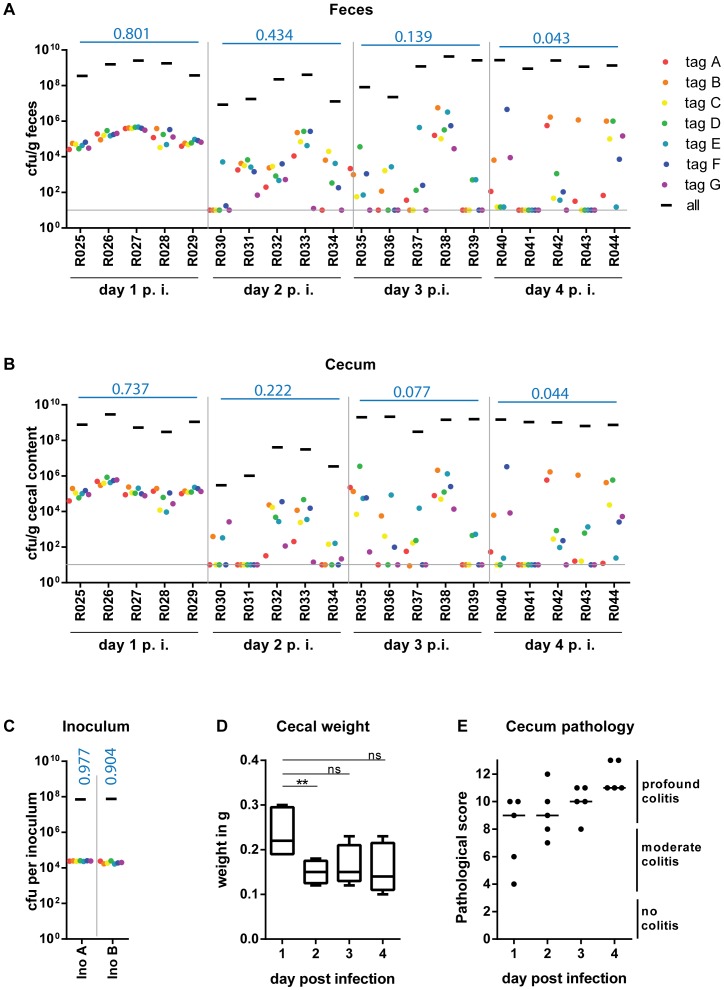
WITS infections reveal severe WITS diversity loss ( = reduction in population evenness) during oral infection of C57BL/6 mice starting at day 2 p.i. Streptomycin-pretreated C57BL/6 mice were gavaged with a mixture of 7 WITS strains in equal ratios, which were further diluted with untagged *S.* Tm^WT^ to a final proportion of 1∶7000. The cfu/g fecal (A) or cecal (B) content of the whole population and the individually tagged strains were monitor daily until day 4 post infection (day 4 p. i.) of each mouse (individual mouse identifiers were plotted on the x-axis). Grey lines depict the detection limit for plating (10 cfu/g). (C) rtqPCR analysis and selective plating of the inoculum used for infection confirmed the even distribution of the seven tagged strain, and the 1000-fold overrepresentation of the untagged *S.* Tm^WT^ strain. (D) To analyze the whole cecal population, the whole cecal content was retrieved. As the cecum shrinks in the course of *S.* Tm^WT^ infection [16], we monitored the total cecal weight over time. (E) At day 4 p.i. all animals were highly inflamed as assessed by a cecal pathology score. Blue numbers indicate the median of the evenness indices of single mice.

Next, we analyzed the time course of changes in the WITS genetic diversity. We employed a “1∶7000” WITS inoculum, as the data above had indicated that this retains high evenness indices for stool samples by day 1 and should allow sensitive detection of changes during the subsequent days. The 1∶7000 inoculum was prepared as described above and we infected streptomycin pretreated C57BL/6 mice (5×10^7^ cfu total, by gavage; 2 independent experiments using a total of 5 mice per group). Total pathogen loads and the WITS in the inoculum, the stool, the cecal content, the mLN, the spleen and the liver were quantified by plating and rtqPCR at day 1, 2, 3 or 4 post infection (Materials and Methods). In line with earlier results, *S.* Tm efficiently colonized the feces and the cecum contents within the first day (10^8^–10^9^ cfu/g; Fig. 2A,B, black bars), elicited pronounced cecum tissue contraction and inflammation (Fig. 2D,E) and the pathogen spread to the mesenteric lymph nodes, the spleen and the liver (S4 Figure, black bars). This verified that the infection had proceeded as expected.

At day 1 p.i., the bacterial populations in the feces and in the cecum content featured evenness indices almost as high as the inoculum (median_feces_ = 0.801; median_cecum content_ = 0.737; each WITS present; Fig. 2 ABC). This was in stark contrast to the *S.* Tm populations in the mLN, spleens and livers, which harbored no WITS at all (S4 Figure; evenness index below detection). In line with earlier work, this indicated that systemic dissemination is partly restricted by a pronounced barrier and that these organs are populated by local growth of a few “founder” bacteria ([6], [29]; not analyzed further in this study). In contrast, the gut luminal population retained all WITS and the high evenness indices indicated that *S.* Tm did not encounter detectable bottlenecks during the transit through the stomach and gastrointestinal tract or the initial colonization of the gut lumen. Based on the 1∶7000 composition of the inoculum, we applied a simple binomial selection model to estimate that the luminal *S.* Tm population size never drops below 2×10^4^ cfu during this initial day of infection (Table 2; Materials and Methods). Please note that this is an estimate of the lower boundary (detectable with WITS-dilutions of 1∶7000 and using 10 mice) and that the actual *S.* Tm population size might in fact be much larger than this.

**Table 2:** Bottleneck estimates.

Bottleneck estimates for different lengths of infection (Fig. 2)			
	Bottleneck estimate	Confidence interval Lower bound	Confidence interval Higher bound
Day 1 p.i., Feces	NA	25265	NA
Day 1 p.i., Cecum	NA	20509	NA
Day 2 p.i., Feces	11265	7328	16752
Day 2 p.i., Cecum	5931	3656	9060
Day 3 p.i., Feces	6932	4360	10452
Day 3 p.i., Cecum	8102	5176	12102
Day 4 p.i., Feces	6932	4360	10452
Day 4 p.i., Cecum	5931	4242	8046

Bottleneck estimates for different inflammatory conditions (Fig. 3&4)			
	Bottleneck estimate	Confidence interval Lower bound	Confidence interval Higher bound
SPI1 deficient mutant	NA	20509	NA
SPI2 deficient mutant	NA	22809	NA
SPI1&2 deficient mutant	NA	23727	NA
SPI2 mutant diluted in WT	7690	5098	11137

Bottleneck estimates for Gr-1 depletion (Fig. 6)			
	Bottleneck estimate	Confidence interval Lower bound	Confidence interval Higher bound
PBS control	5193	3287	7761
Gr-1 depletion	NA	20509	NA

Bottleneck estimates and 95% confidence intervals for different inflammatory conditions and during different lengths of inflammation.

NA indicates that there is no proper estimate. This happens mainly in the cases where no WITS are lost. In these cases only a lower bound is applicable.

Reduced evenness indices were observed in the cecum lumen and the feces by day 2 p.i. and evenness further declined until day 4 p.i. (median_feces_ = 0.434→0.043; median_cecum content_ = 0.222→0.044; Fig. 2 A, B). By day 4 p.i., most mice had “lost” one or more WITS from the cecal contents and the feces (detection limit: 10 cfu/g). This indicated that some type of barrier may restrict the gut luminal *S.* Tm population from day 2 on.

A simple estimate of the number of bacteria that can cross this barrier can be done from the data on the presence or absence of WITS at day 4 after inoculation at a dilution of 1∶7000 (see Materials and Methods). In combination with the 1∶7000 dilution experiment shown in Fig. 1D, we had a total of 10 animals infected for 4 days. In these mice, 30 of the 70 WITS were lost from the cecum lumen at day 4 (Fig. 1D, Fig. 2B). Assuming that each WITS succeeds in crossing the barrier according to a binomial process with probability 1/7000, we estimated a bottleneck size of 5931 bacteria with a 95% confidence interval ranging from 4242 to 8046 bacteria (Materials and Methods).

Within each animal, the WITS distribution patterns of the cecal content and the feces were strikingly similar, in particular at days 3 and 4 p.i. (R^2^ = 0.854 or 0.961, respectively). Therefore, these populations are linked and fecal samples can be used to monitor the cecum luminal pathogen population at days 3 and 4 p.i. Conceptually, this suggests that the cecum lumen may produce (or seed) the pathogen population shed in the feces.

In conclusion, these data established that the gut luminal *S.* Tm population is restricted by a significant barrier by day 2 p.i. and that this bottleneck can be detected via the reduced evenness index of the WITS-diversity. It is interesting to note that the cecum luminal pathogen density transiently dropped at day 2 p.i. and was as low as 10^5^–10^6^ cfu/g in two of the mice (Fig. 2B, mouse #R030 and R031, black bars). Thus, a transient reduction of the total gut luminal *S.* Tm population size (a “population bottleneck”) may contribute to the barrier restricting the gut luminal *S.* Tm population. The nature of this barrier remained to be determined.

### A SPI-1 SPI-2 double mutant does not encounter a bottleneck in the gut lumen

**Figure 3:**
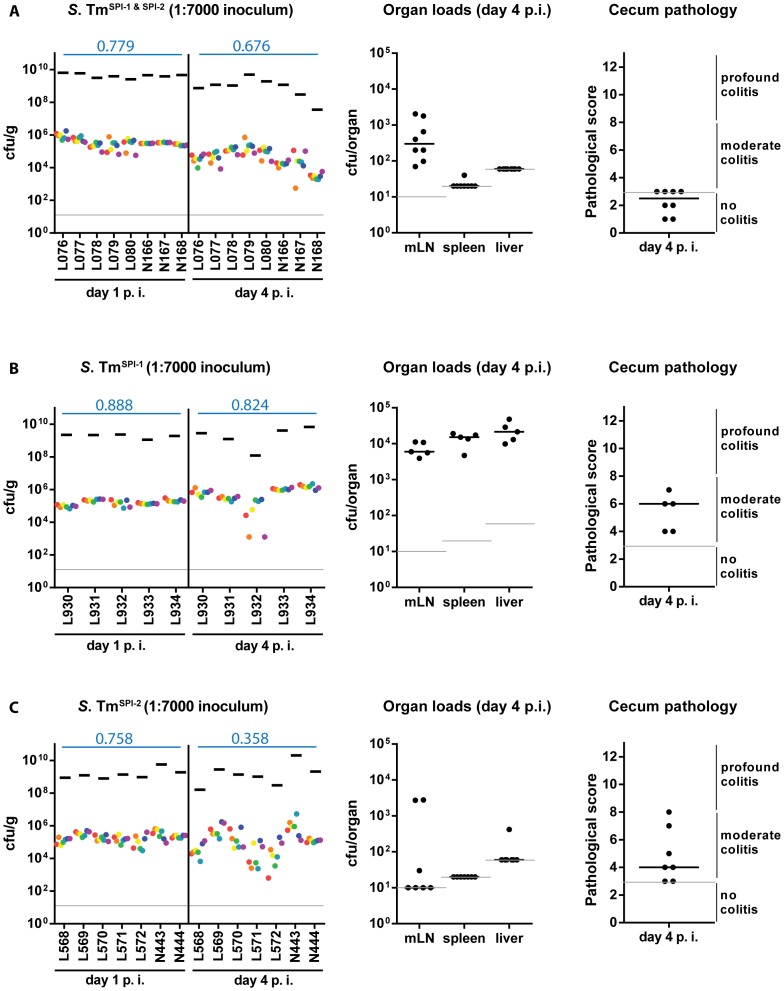
WITS diversity loss is inflammation-dependent. Streptomycin-pretreated C57BL/6 mice were orally infected with a WITS cocktails in the dilution 1∶7000 in different strain backgrounds: (A) *S.* Tm^SPI-1 & SPI-2^, (B) *S.* Tm^SPI-1^ and (C) *S.* Tm^SPI-2^ and WITS compositions throughout the infection process were monitored by rtqPCR. For each conditions, organ loads and cecum pathology at day 4 p. i. were monitored. Grey lines depict the detection limit for plating (10 cfu/g for feces, cecal content and mLN, 20 cfu/organ for spleen and 60 cfu/organ for liver). Blue numbers indicate the median of the evenness indices of single mice.

Next, we analyzed if mucosal inflammation contributes to the gut luminal population bottleneck. This seemed plausible, as inflammatory responses can (at least in tissues) eliminate pathogens via an elaborated arsenal of antimicrobial mechanisms [26]. Gut luminal colonization without the elicitation of mucosal inflammation can be achieved by using “avirulent” *S.* Tm mutants deficient in the SPI-1 and SPI-2 type III secretion systems [18], [19]. To this end, we constructed “avirulent” WITS by P22 phage transduction of the chromosomal tags into an *invGssaV* mutant (WITS^SPI-1 & SPI-2^; Materials and Methods) and prepared a “1∶7000” inoculum by mixing with an untagged *S.* Tm^SPI-1 & SPI-2^ strain as described above. Streptomycin pretreated C57BL/6 mice were infected (5×10^7^ cfu total, by gavage) and we analyzed pathogen loads in the stool, the WITS diversity in stool samples, gut pathology and pathogen loads at systemic sites (Fig. 3A). As expected, the *S.* Tm mutant yielded only minimal colonization of the spleen and the liver, reduced pathogen loads in the mLN, reduced total gut luminal loads by day 4 p.i. and no signs of overt mucosal inflammation by day 4 p.i. (pathoscore 2.5+/−1). The WITS evenness index in the stool was high at day 1 p.i. and only slightly reduced by day 4 p.i. (median = 0.799→0.676; Table 1). Conceivably, the slightly reduced evenness index detected at day 4 p.i. might be attributable (at least in part) to a reduced total *S.* Tm population size, as loads in the stool reached very high levels at day 1 p.i. (10^9^–10^10^ cfu/g), but decreased by about 10-fold to 10^8^–10^9^ cfu/g by day 4 p.i. (Fig. 3A, black bars). This is in line with earlier work which established that, in the absence of gut inflammation, the re-growing microbiota can outcompete avirulent *S.* Tm mutants lacking functional SPI-1 and SPI-2 type III secretion systems [20]. Nevertheless, the WITS-diversity was only slightly reduced by day 4 p.i., suggesting that the tight bottleneck observed in wild type *S.* Typhimurium infections is attributable to mucosal inflammation.

### SPI-1 is more important than SPI-2 for eliciting WITS-diversity loss

Next, we tested the individual contributions of SPI-1 and SPI-2 to WITS-diversity loss. For this we used *S.* Tm mutants lacking either a functional SPI-1 TTSS (*S.* Tm^SPI-1^, *ΔinvG*; strong defect in eliciting gut inflammation at day 2 p.i.) or a functional SPI-2 TTSS (*S.* Tm^SPI-2^, *ssaV::cat*; slight defect in eliciting gut inflammation at day 2 p.i.), which do efficiently colonize the gut lumen of streptomycin pretreated mice, but elicit reduced levels of mucosal inflammation on day 2 p.i. [16], [18], [19], the time when the gut luminal bottleneck is observed. The respective WITS were constructed by P22 phage transduction thus yielding WITS^SPI-1^ (*ΔinvG* background; [6], [34], [35]) and WITS^SPI-2^ (*ssaV::cat* background; [6],[35] Materials and Methods) and mice were infected with “1∶7000” inocula and analyzed as described for Fig. 1.

As expected, the SPI-1 mutant efficiently colonized the spleen, the liver and the mLN and elicited moderate mucosal inflammation by day 4 p.i. (Fig. 3B). In line with earlier data, the mucosa featured a “patchy” distribution of inflammatory foci typical for the “SPI-2 mediated” disease [19], [36] which was even at day 4 p.i. significantly less pronounced than the inflammation elicited by wild type *S.* Tm (compare Fig. 1E, 2E with Fig. 3B). The stool loads remained high (10^9^–10^10^ cfu/g) throughout the experiment and we observed at most a slight decrease of the evenness index (median = 0.888→0.824; Table 1).

In line with the key role of SPI-2 in systemic infection [19], [37], the SPI-2 mutant did not efficiently colonize the spleen, liver and the mLN, but elicited mucosal inflammation (Fig. 3C). Please note that the “SPI-1 mediated” mucosal inflammation elicited by this strain is much more pronounced than the “SPI-2 mediated” inflammation at day 2 p.i. [19]. In the present experiment, we analyzed the gut inflammation at a much later time point, i.e. day 4 p.i. Here, inflammation was much milder than the pathology elicited by wild type *S.* Tm (compare Fig. 1E, 2E with Fig. 3C; [6], [19]). Strikingly, the WITS^SPI-2^ population featured a moderate drop of the evenness index between days 1 and 4 p.i. (median = 0.758→0.358; Table 1). These data are in line with the notion that mucosal inflammation (i.e. the degree of inflammation at day 2 p.i.) may drive the WITS-diversity loss and suggest that the grade of inflammation may dictate (at least in part) barrier efficiency.

### Mixed infection experiments verify that mucosal inflammation is required for WITS-diversity loss

**Figure 4:**
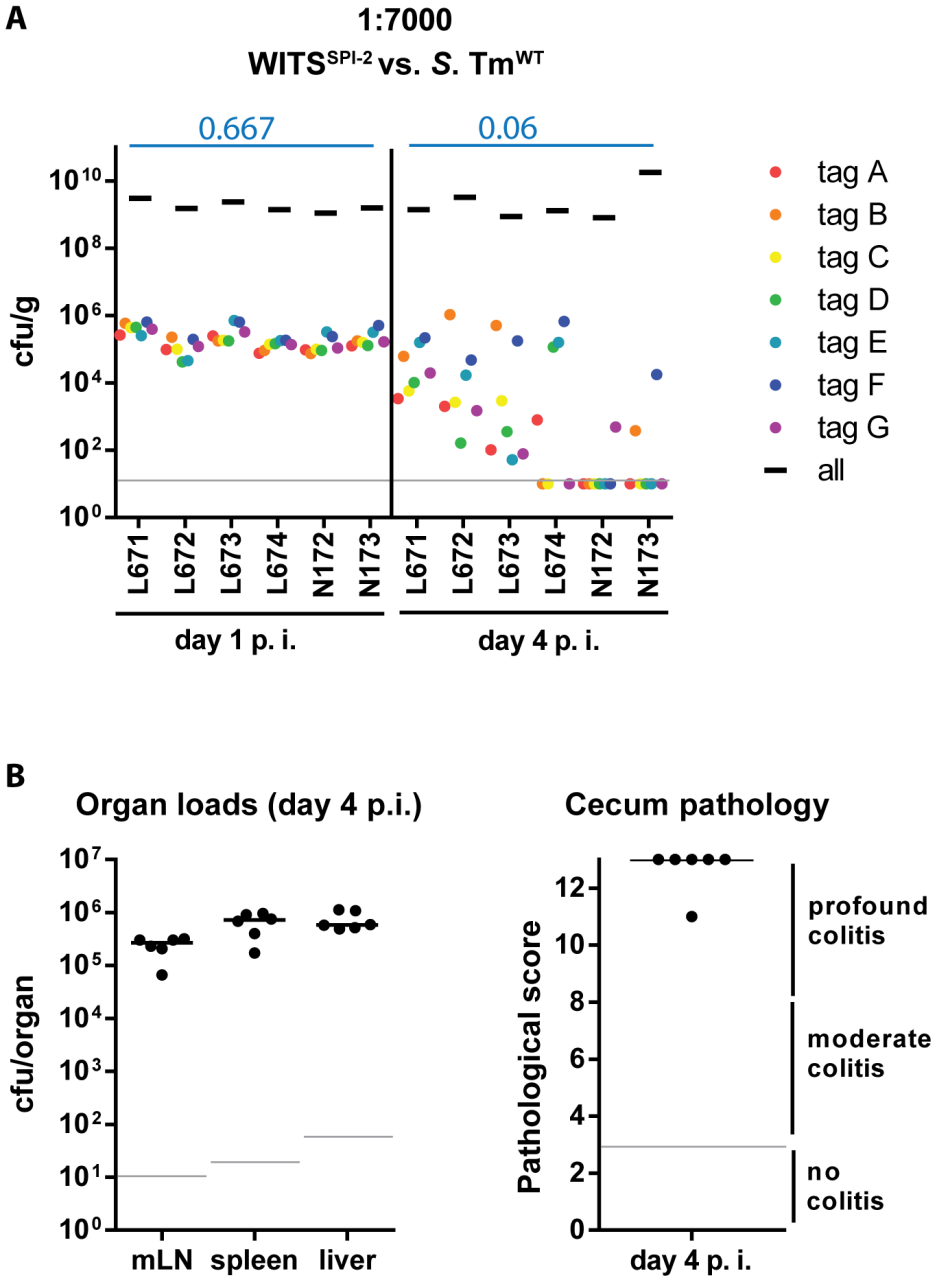
Severity of inflammation, not genetic background of the tagged strains, determines WITS diversity loss. (A) To exclude a strain-intrinsic property of the SPI-2 WITS to be responsible for the decrease in WITS diversity loss observed in the *S.* Tm^SPI-2^ background, we tested a chimeric situation in which inflammation was caused by *S.* Tm^WT^ but WITS-tags were in a SPI-2 deficient background. Experiments were performed using a WITS cocktail in a dilution of 1∶7000. Grey lines depict the detection limit for plating (10 cfu/g). Blue numbers indicate the median of the evenness indices of single mice. (B) Organ loads and cecum pathology at day 4 p.i. confirmed the “WT-like” severity of the infection.

To study the effect of mucosal inflammation on strains incapable of eliciting disease one can perform a modified version of our mixed inoculum experiments. The inflammation elicited by wild type *S.* Tm will affect all bacteria present in the gut lumen (i.e. wild type and mutant [20], [38]–[42]). Thus, we performed a variant of the assay described above to verify that the bottleneck is indeed affected by the grade of the inflammatory response (not genetic predisposition of the *S.* Tm strain). In particular, we wanted to assess the evenness index of WITS^SPI-2^ in the face of full blown mucosal inflammation elicited by wild type *S.* Tm. This was of particular interest, as WITS^SPI-2^ can colonize the gut lumen, but fails to efficiently grow in the mucosal lamina propria and at systemic sites [19]. Thus, using WITS^SPI-2^ would help to exclude possible re-seeding from such sites and therefore allow focusing on the gut luminal pathogen population. To this end, we mixed WITS^SPI-2^ with an excess of wild type *S.* Tm [6], [38]. Streptomycin pretreated C57BL/6 mice were infected for 1 or 4 days with a “1∶7000” inoculum composed of WITS^SPI-2^ and untagged *S.* Tm^WT^ and we analyzed the infection as in Fig. 1. Pathogen organ loads (i.e. WITS^SPI-2^ plus untagged *S.* Tm^WT^) and cecum pathological scoring confirmed an overall “wild type”-like intestinal disease progression during this mixed infection experiment (Fig. 4A,B, compare to Fig. 2E; black bars). Remarkably, in this experiment, the WITS^SPI-2^ evenness index dropped much further than in infections performed with WITS^SPI-2^ alone by day 4 p.i. (median = 0.06; Fig. 4A, compare to Fig. 3C). In fact, the evenness index at day 4 p.i. was strikingly similar to that of wild type WITS (median = 0.033 and 0.044; see Fig. 1D and 2A,B; Table 1). We therefore conclude that the bottleneck is determined by the degree of inflammation, not by the genetic background of the tagged subpopulation.

### The size of the cecum luminal *S.* Tm population is diminished at day 2 post infection

**Figure 5:**
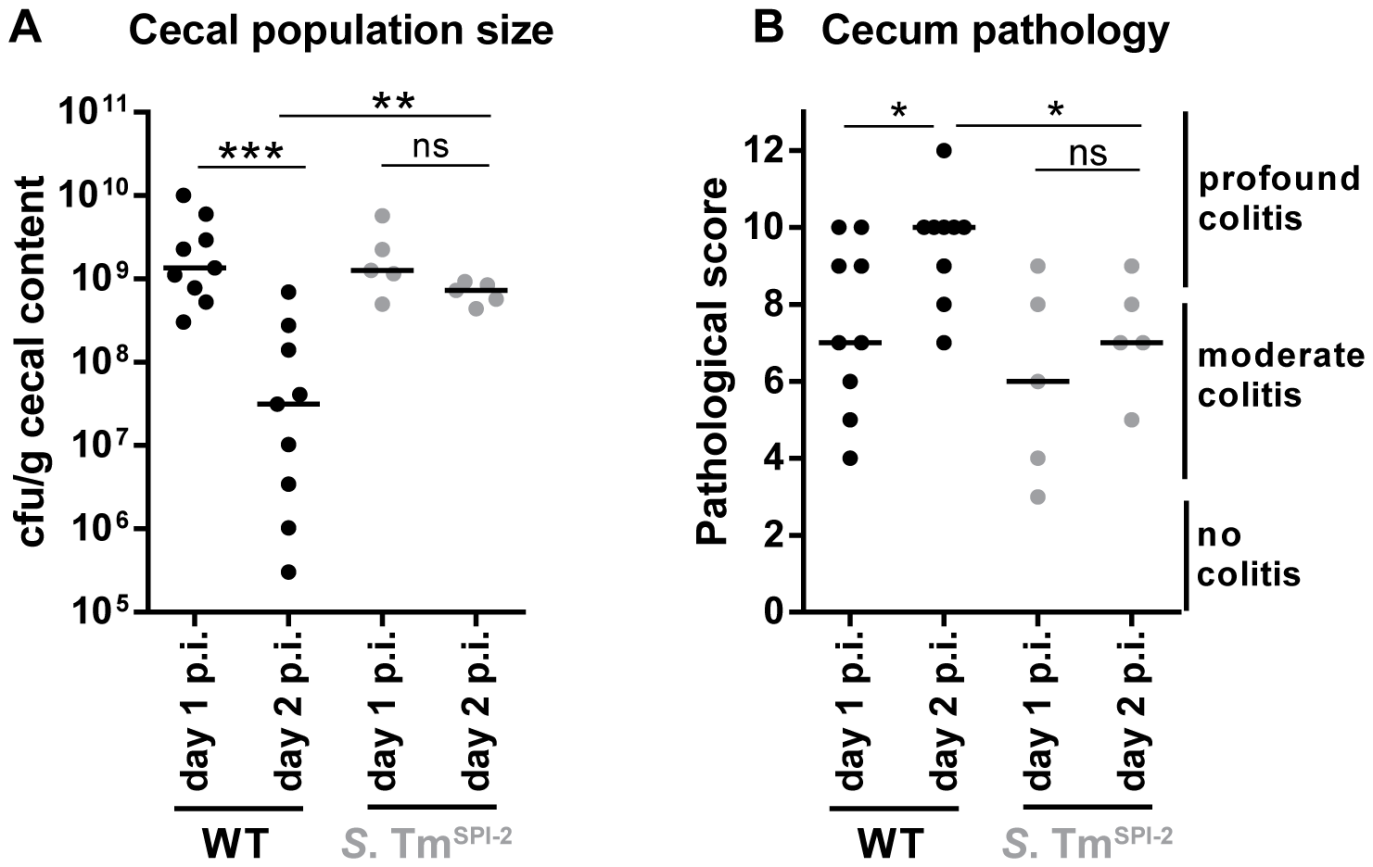
Decrease in cecal population size between day 1 and day 2 p.i. only occurs during *S.* Tm^WT^ induced inflammation, but not during inflammation in a SPI-2 deficient background. Streptomycin-pretreated C57BL/6 mice were orally infected with *S.* Tm^WT^ or *S.* Tm^SPI-2^, respectively. Mice were sacrificed at day 1 or day 2 p.i. and bacterial population size (A) and cecum pathology at the day of sacrifice (B) were plotted. ns = not significant (P≥0.05), * P<0.05, ** P<0.01, *** P<0.001; Mann-Whitney U test.

Reduced WITS-evenness can be explained by a drastic reduction in the cecal *S.* Tm population size and a subsequent (oligo-clonal) regrowth of the population. In fact, transient reductions of the cecal and fecal population densities at day 2 p.i. have been observed repeatedly (though never followed up in detail), over the past decade ([20]; Fig. 2A,B). To specifically analyze the population size at this critical time point, we monitored changes in cecum luminal *S.* Tm population size by sacrificing *S.* Tm^WT^-infected mice at day 1 and at day 2 p. i. and by determining the respective pathogen loads. In line with earlier data, the mice featured moderate colitis by day 1 and pronounced colitis by day 2 p.i. (Fig. 5B). The pathogen densities in the cecum lumen were high at day 1 p.i. (≈10^9^ cfu/g), but dropped significantly by day 2 (median = 3×10^7^ cfu/g, Fig. 5A). Strikingly, the gut luminal pathogen density featured pronounced animal-to-animal variation. In some animals, the pathogen density dropped down to 3×10^5^ cfu/g. Without further information, these data cannot tell whether the dip in the gut luminal pathogen density is more pronounced in some animals than in others. Alternatively, equivalent dips may occur in all mice however with slightly different kinetics. Nevertheless, the data suggests that a transient dip in the cecum luminal population size may contribute to the drop in WITS evenness at least in some of the mice.

If pathogen-triggered inflammation was responsible for the dip at day 2 p.i., cecal *S.* Tm densities should remain higher in cases of reduced mucosal inflammation. To test this hypothesis, we infected mice with S. Tm^SPI-2^ and compared cecal population sizes at day 1 and day 2 p. i. Indeed, under these conditions cecal *S.* Tm loads remained equally high from day 1 to day 2 (Fig. 5).

Taken together, our observations suggest that *S.* Tm^WT^ triggered inflammation can inflict a transient population bottleneck at day 2 p. i. and that this contributes at least in part to the reduced evenness index, observed in our WITS experiments.

### Depletion of Gr1^+^-cells alleviates the bottleneck

Thus far, our data suggested that the grade of the mucosal inflammation is a key determinant of the bottleneck at day 2 p.i. However, the mechanisms explaining this barrier to infection remained to be identified. One hallmark of *S.* Tm induced inflammation is the tissue infiltration by granulocytes, their transmigration into the gut lumen and the formation of characteristic crypt abscesses [15]. As granulocytes can attack bacteria by bactericidal mechanisms such as phagocytosis, release of antimicrobial substances and formation of neutrophil extracellular traps (NETs) [43], they might contribute to the gut luminal bottleneck. However, to the best of our knowledge, it has not been assessed previously, whether granulocytes can have bactericidal activity upon transmigration into the gut lumen.

**Figure 6:**
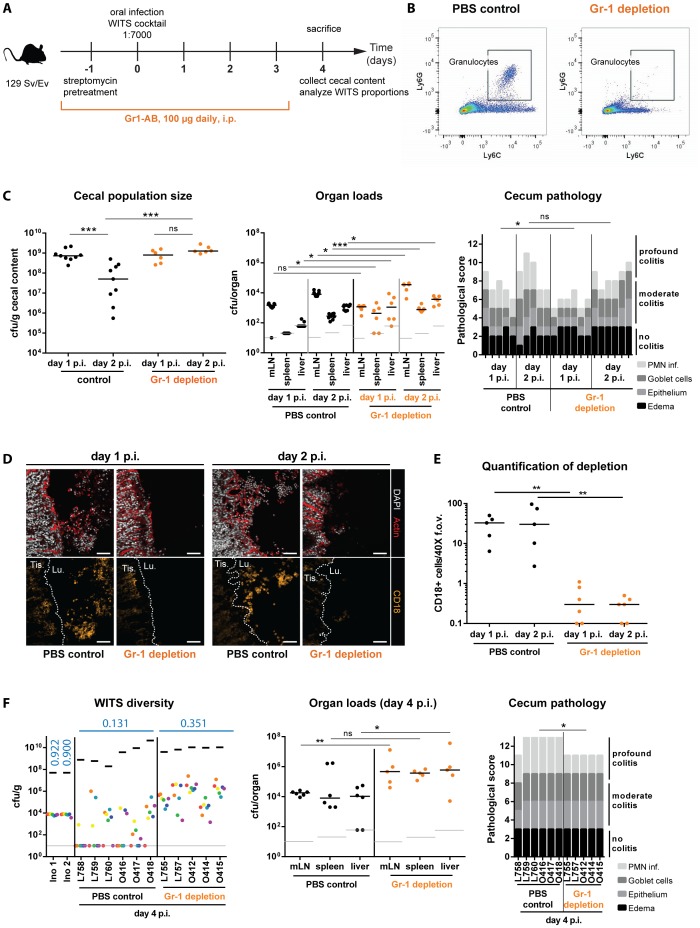
Depletion of Gr1^+^- cells reduces WITS diversity loss and prevents the cecal population crash between day 1 and day 2 p.i. (A) Schematic overview of the experimental setup. Starting with streptomycin-pretreatment one day prior to infection with the *S.* Tm^WT^ WITS cocktail in a dilution of 1∶7000, Gr1^+^-cells (mostly neutrophilic granulocytes) were depleted by daily i.p. injection of 100 µg of anti-mouse-Gr1-antibody. The control group was injected with an equal volume of PBS. (B) Successful reduction in Ly6C^+^ Ly6G^+^ cells in the murine blood was verified daily using a flow cytometry assay. A representative blot is given in panel B. (C) The cecal *S.* Tm population size was monitored in the Gr1^+^-cell depleted mice and the control group and organ loads and cecum pathology of respective animals were assessed. (D) CD18 immunofluorescent staining of cecal section from Gr1^+^-cell depleted mice and PBS treated control groups confirmed reduction of granulocytes in the course of the experiment. Scale bars: 50 µm (E) Quantification of luminal granulocytes from tissue sections. (F) At day 4 p.i. animals were sacrificed and the population composition in cecal contents were analyzed by rtqPCR. Organ pathogen loads and cecum pathology at day 4 p.i. indicate systemic pathogen spread and high intestinal inflammation. ns = not significant (P≥0.05), * P<0.05, ** P<0.01, *** P<0.001, Mann-Whitney U test. Grey lines indicate detection limits. Blue numbers indicate the median of the evenness indices of single mice.

To test if granulocyte-mediated killing contributes to the gut luminal bottleneck, we employed a cell depletion strategy. Initially, we depleted granulocytes during *S.* Tm^WT^ infection using an α-Gr1 antibody (100 µg i.p., once per day; Materials and Methods; Fig. 6A) which binds to Ly6G on neutrophilic granulocytes (PMN) and to Ly6C, a marker expressed on neutrophils and on dendritic cells, some subpopulations of lymphocytes and some monocytes [44]. For this experiment, we used 129SvEv mice (*Nramp*/*slc11α1* ^+/+^) as their monocytes can control *S.* Tm growth at systemic sites much more efficiently than those from *slc11α1* ^−/−^ C57BL/6 mice [45], [46]. This allowed infecting Gr1^+^-depleted mice for 4 days without compromising the viability of the animal (Fig. 6A).

First we analyzed the effect of granulocyte depletion on the cecum luminal pathogen density at day 2 p.i. Gr1^+^-cell-depleted mice, and PBS-treated control animals were pretreated with streptomycin and infected for 1 or 2 days with *S.* Tm^WT^. The efficiency of granulocyte depletion was verified by flow cytometry (CD45^+^, Ly6G^+^, Ly6C^+^ cells; Fig. 6B) of murine blood. The detailed effect of Gr-1 neutralization on inflammatory myeloids in the cecal mucosa of 129SvEv mice revealed that the procedure depleted not only granulocytes, but also other phagocyte populations (S5 Figure, S1 Text). Pathogen loads in the mLN, the spleens and the livers did rise in both groups by 10–100 fold between day 1 and day 2 p.i. We detected (at most) slightly elevated tissue loads in the Gr1^+^-depleted mice, while both groups featured equivalent *S.* Tm^WT^ loads in the cecal lumen at day 1 p.i. (Fig. 6C). Strikingly, the cecum luminal pathogen loads remained high at day 2 p.i. in the Gr1^+^-depleted mice, but plummeted in the PBS controls. This went along with reduced gut luminal granulocyte (CD18^+^) numbers in the Gr1^+^-depleted mice (Fig. 6D,E) and suggested that granulocytes may indeed affect the bottleneck.

A second experiment was performed to assess effects on the WITS evenness index. Gr1^+^-depleted mice, and PBS-treated control animals were pretreated with streptomycin and infected for 4 days with a “1∶7000” inoculum of *S.* Tm^WT^ WITS as described for Fig. 2. In line with earlier work, the Gr1^+^-depleted mice featured increased organ loads in the mLN, the spleens and the livers [47]. This verified that granulocytes indeed significantly limit systemic infection. In contrast, cecum luminal total *S.* Tm^WT^ densities did not differ between both groups (Fig. 6F, left panel, black bars). Strikingly, the WITS evenness index remained much higher in the Gr1^+^-depleted mice, as compared to the PBS controls (Fig. 6F, Table 1). In a complementary, yet more specific strategy, we applied a combination of α-Ly6G and α-G-CSF antibodies to selectively reduce granulocytes but spare monocytes from depletion [48], [49]. Again, the depleted animals featured higher WITS evenness indices than the control group treated with the respective isotype controls of both antibodies (S6 Figure, S1 Text). These data supported our hypothesis that Gr1^+^ cells (mainly granulocytes) form a major bottleneck for the gut luminal *S.* Tm population by day 2 p.i. However, these results raised the question whether this outcome is a direct effect of the bactericidal activity of granulocytes (e.g. NO/ROS production) or an indirect effect (e.g. reduction of granulocyte-released cytokines or antimicrobial proteins). First, we examined WITS diversity loss in *Nos2*-deficient mice to clarify the requirement of inflammation-mediated NO production in reducing luminal *S.* Tm. As Nos2 was shown to be dispensable for controlling *S.* Tm during the initial days of infection [50], we hypothesized that the *Nos2*-deficient mice should restrict gut luminal *S.* Typhimurium loads as efficiently as the wild type animals. In line with this, the WITS evenness indices in *Nos2^−/−^* mice and the littermate controls did not differ at day 3 p.i. (S7A Figure, S2 Table) suggesting that NO is not the predominant cause for the WITS diversity loss.

Next, we tested the contribution of oxidative burst in the reduction of WITS evenness. As NADPH-oxidase is a key antimicrobial factor of granulocytes [43] at systemic sites and in the intestinal tissue [51] and as these cells do transmigrate in large numbers into the lumen of the inflamed gut, we speculated that *Cybb^−/−^* mice might be incapable of restricting gut luminal pathogen loads in the inflamed gut. Strikingly, WITS evenness in the NADPH-oxidase deficient (*Cybb^−/−^*) mice at day 3 p.i. was increased compared to a heterozygous control group (S8A Figure, S2 Table) pointing to a possible involvement of direct ROS-mediated killing to the bottleneck effect. However, interpretation of WITS analysis in the *Cybb^−/−^* mice is limited by a high *S.* Tm colonization at systemic sites up to levels where WITS-tagged strains are found systemically (S8B Figure). As possible re-seeding events of these strains into the gut lumen may compromise the unequivocal interpretation of our WITS-diversity analysis, these results should be taken with caution.

Taken together, the gut luminal *S.* Tm population bottleneck is mostly caused by granulocytes, possibly via a direct ROS-mediated killing mechanism. However, our data do not exclude the contribution of additional factors to this bottleneck effect.

## Discussion

To identify barriers limiting *S.* Tm in the gut lumen, we have performed mixed inoculation experiments. During the first day, the pathogen density rose to ≈10^9^ cfu/g in the large intestinal lumen without encompassing obvious bottlenecks. In the case of wild type *S.* Tm infection, cecum colonization was accompanied by pronounced mucosal inflammation which arises during the first day (8–12 h p.i.; [15], [52]) and lasts for four days (or longer; [46], [53]). By day 2 p.i., the inflammatory response dramatically reduces the gut luminal pathogen density. This inflammation-inflicted, granulocyte-dependent bottleneck transiently restricts the gut luminal *S.* Tm population to about 6000 cfu. By days 3 and 4, the pathogen density rises again to ≈10^9^ cfu/g of gut content. These data establish that mucosal inflammation can represent a barrier limiting the gut luminal colonization by the pathogen.

Our findings significantly extend earlier work on *S.* Tm growth in the lumen of the inflamed gut. That earlier work had indicated that the pathogen subverts the inflammatory milieu in the gut lumen for its own advantage, i.e. for outcompeting the resident microbiota (reviewed in [15], [54], [55]). Granulocytes infiltrating the inflamed mucosa and transmigrating into the gut lumen are thought to contribute in several different ways i.e., by i) providing/regenerating terminal electron acceptors (tetrathionate, nitrate) used by *S.* Tm, thereby fuelling anaerobic respiration and fast growth of the pathogen (not the commensals, which are mainly fermenters [24], [25]); by ii) releasing lipocalin-2, an antimicrobial peptide blocking siderophore-mediated iron acquisition by *E. coli*. Interestingly, *S.* Tm is endowed with the *iroBCDEN* variant of such system which can bypass the lipocalin-2-mediated restriction [23]; by iii) releasing calprotectin which sequesters Zn^2+^, an essential micronutrient, from the gut lumen. This restricts microbiota growth, while *S.* Tm can bypass this restriction using a high affinity zinc transporter (*znuABC*; [22]). Furthermore, *S.* Tm is endowed with enzymes inactivating PMN-derived reactive oxygen and nitrogen compounds [56]–[58]. Overall, these earlier findings indicated that granulocyte activity (and mucosa inflammation in general) foster pathogen growth in the intestine and prevent growth of the resident microbiota. However, the quantitative contribution of inflammation-mediated fuelling versus restriction of *S.* Tm growth in the gut lumen had remained unclear. Our findings demonstrate that the gut luminal *S.* Tm population is highly vulnerable to the inflammatory response in particular at day 2 p.i. We conclude that the size of the pathogen population in the inflamed gut is controlled by a fine balance between inflammation-fuelled pathogen growth (and competition against resident microbiota) and inflammation/granulocyte-inflicted losses to the pathogen population.

How many *S.* Tm cells survive the bottleneck in the gut lumen? Our best estimate is that 6000+/−2000 bacteria survive this dip at day 2. This value is obtained from a simple binomial selection model fitted to the data on the detectability (presence/absence) of WITS at day 4 for the 1∶7000 dilution. This model assumes that there are no fitness differences between WITS and that the population bottleneck is effective for only a short time. The classical cfu-plating revealed reduced, but highly variable pathogen population sizes at day 2 p.i. (median = 2×10^6^ cfu/g). In a few mice, we detected as few as 3×10^5^ cfu/g of cecum content by day 2 p.i. This corresponds to a total population size of 15000 bacterial cells, considering that the infected cecum (tissue+lumen) has a total volume of no more than 200 µl, and that the cecal luminal volume might range at approx. 50 µl at this stage. The high variance of the cfu-data measured at day 2 p.i. and the similarity of the evenness-indices across animals suggest that the pathogen population may be reduced for no more than a few hours before net re-growth occurs. Using 24 h sampling intervals, classical cfu-plating would therefore miss the low in most mice, in particular if the time-course would differ from animal to animal. Therefore, we conclude that the bottleneck allows the survival of just 6000 bacteria. Thus, the mucosal inflammation is a strikingly efficient host defense, reducing the total cecum luminal population by about 10^5^–10^6^ fold (10^8^ cfu per cecum at day 1 p.i. to 6000 bacteria by day 2). This is almost as efficient as chemical disinfectants (defined as reducing bacterial survival by ≥10^6^-fold; [59]) and far more effective than many other barriers, e.g. the IFNγ or inflammasome mediated restriction of systemic spread (≈10-fold restriction by day 2 p.i.; [60], [61]) or the gut luminal growth defect inflicted by adaptive sIgA response directed against the LPS O-sidechain (≈10-fold; [53], [62]) limiting *S.* Tm loads at different stages of an infection. In conclusion, the mucosal inflammation severely reduces the gut luminal *S.* Tm population by day 2 p.i. However, this is not quite sufficient to yield sterilizing protection and to prevent subsequent pathogen-regrowth.

In this work, the importance of granulocytes in controlling *S.* Tm within the gut lumen is re-emphasized and extended. Already in the early studies of streptomycin pretreated mice, neutrophil infiltration was detected in the cecal mucosa by 10 h p.i. [63]. Similarly, granulocytes are prominent in the infected mucosa of cows and man [64], [65], they transmigrate into the gut lumen and form the archetypical “crypt abscesses”, i.e. densely packed clusters of granulocytes located within the intestinal crypts [66]–[69]. By extension, these observations suggest that granulocyte-mediated growth and restriction might be of general importance also in other hosts including the human patient.

During the first day of infection, granulocytes might also serve as a host cell permissive for intracellular growth (or at least survival) of the pathogen. In the streptomycin mouse model, gut luminal neutrophils harbored significant numbers of *S.* Tm by 20 hours p.i. and most of these bacterial cells were viable, as judged from the expression of *iroBCDE*- and *ttss-2* driven GFP reporters [70]. Other studies have also indicated that *S.* Tm can survive in murine neutrophils [70]–[72]. The lack of a bottleneck during the first day of infection would be in line with this. Our data suggest that the barrier detected by day 2 p.i. is attributable to some quantitative or qualitative change in the inflammatory response and/or the granulocyte activity, e.g. by modulating NADPH-oxidase dependent defenses. Identifying the underlying mechanism controlling the granulocyte-inflicted bottleneck will be of interest for future work and might identify strategies for bolstering the gut luminal barrier. Still, our data suggest that pronounced granulocyte migration into the gut lumen can have detrimental effects on the *S.* Tm population, at least by day 2 p.i. Furthermore, our data provide first hints that ROS might play a central role in decimating luminal *S.* Tm, despite of the low oxygen-availability in the gut lumen and the fact that *S.* Tm expresses three catalases and three periplasmic Cu,Zn superoxide dismutase enzymes [73]. Thus, the interaction of *S.* Tm with granulocytes in the gut lumen is clearly important, but more complex than previously anticipated.

In summary, investigating mixed inocula allows precious insights into colonization strategies and host defenses limiting infection. The population bottlenecks identified by such approaches may represent attractive points for therapeutic interventions, as pathogen populations are already reduced to a minimum.

## Materials and Methods

### Bacterial strains

The wild type strain SB300 is a clone of *S.* Tm SL1344 and the SPI-1 and SPI-2 deficient mutant strains (*S.* Tm^WT^, *S.* Tm^SPI-1^, *S.* Tm^SPI-2^), as well as their isogenic tagged strains have been described previously [5], [6], [34], [35], [74]. WITS-tags were introduced into *S.* Tm^SPI-1 & SPI-2^ (SL1344 *invGssaV*; [17] and into *S.* Tm^SPI-2^ (SL1344 *ssaV::cat*; [35]) by P22 phage transduction and subsequent selection on kanamycin. The presence of the correct WITS-tag was confirmed by PCR using tag-specific primers (see S1 Table).

### Mouse lines

Wild type C57BL/6 mice were bred and kept under specified pathogen free (SPF) conditions in individually ventilated cages at the Rodent Center RCHCI (ETH Zurich). They harbor a complex microbiota and are called “conventional” mice. For reliable and efficient infection with *S.* Tm, streptomycin pretreatment of these mice was performed in order to overcome colonization resistance in all experiments described herein [16]. 129 Sv/Ev mice are Nramp1(+/+) (Slc11α1(+/+)) mice which can control systemic *S.* Tm infection with low bacterial loads at systemic sites but develop acute *S.* Tm colitis upon streptomycin pretreatment [46]. 129 Sv/Ev mice are specified pathogen free animals with a complex microbiota. *Cybb^−/−^* (B6.129S-Cybbtm1Din/J; C57BL/6 background) and *Nos2^−/−^* (B6.129P2-Nos2tm1Lau/J; C57BL/6 background) were bred at the Rodent Center HCI (ETH Zurich, Switzerland). Both mouse lines have been described previously [75], [76].

### Animal infection experiments

Animal infection experiments were performed in 8 to 12 week old mice as described previously [16]: C57BL/6 mice or 129 Sv/Ev mice were pretreated with 20 mg streptomycin 24 hours prior to infection. For infection, bacteria were grown for 12 h in 0.3 M NaCl supplemented LB medium containing the appropriate antibiotic(s), diluted 1∶20 and sub-cultured for 4 h in the same medium without supplementation of antibiotics. Tagged and un-tagged strains were mixed as indicated, washed twice with PBS and mice were infected with 5×10^7^ bacteria by gavage. Each animal was kept in a single cage to avoid transmission between mice. Animals were sacrificed at day 4 p.i. by cervical dislocation. Freshly collected fecal pellets or whole cecal content were harvested, homogenized in 500 µl PBS with steel balls in a Tissue Lyser (Qiagen) for plating to determine the total population size. 250 µl of homogenized feces or cecal content were used to inoculate an over night culture in LB supplemented with 50 µg/ml kanamycin to select for tagged strains. Bacteria from 1 ml of this over night culture were harvested and frozen for genomic DNA extraction. HE-staining of cryo-embedded tissues and subsequent pathoscoring for granulocyte infiltration was performed as described previously [16]. To test for selection of beneficial mutations which might explain the variation in the subpopulations at day 4 p.i., we re-isolated dominant WITS-tagged bacteria from cryo-embedded tissues by plating on kanamycin. Untagged strains were isolated in the same way, but were selected for growth on streptomycin and remained kanamycin sensitive. Competitive infection experiments to test for increased fitness of re-isolated strains were performed by infecting streptomycin-pretreated mice with equal amounts of *S.* Tm^WT^ or a WITS-tagged *S.* Tm^WT^ strain and the isolates. By selective plating, population sizes of both were determined and a competitive index was calculated by dividing the ratio of the isolate to the background strains by the ratio of both strains in the inoculum.

### WITS quantification

Genomic DNA from enrichment cultures was isolated with the QIAamp DNA Mini Kit (Qiagen, Cat. NO. 51306). rtqPCR analysis with FastStart Universal SYBR Green Master (Roche) was performed using primers and temperature profiles as described previously [5]. The population size of each tagged strain was calculated by multiplying the number of kanamycin resistant cfu/g recovered *S.* Tm with the ratio of WITS determined by rtqPCR.

### Depletion of Gr-1^+^-cells

The monoclonal anti-Gr1 antibody NIMP-R14 is highly specific for the murine epitopes Ly6G and Ly6C and was shown to selectively deplete mouse neutrophils [77]. For depletion of Gr-1^+^-cells during *S.* Tm^WT^ infection, we injected 100 µg of anti-Gr1 i.p. on a daily basis, starting 24 h prior to infection. Granulocyte depletion was assessed daily by analysis of peripheral blood (and lamina propria after sacrificing the mice) in a flow cytometry assay using the following staining antibodies: anti-CD11b-PECy7 (clone M1/70), anti-Ly6C-FITC (clone AL-21), anti-Ly6G-450V (clone 1A8) and anti-CD45.2-APC (clone 104). NIMP-R14 hybridoma was a kind gift of Dr. Nancy Hogg (Cancer Research UK, London, U.K.) and antibodies for flow cytometry were purchased from BioLegend.

### Immunofluorescence microsopy of fixed tissue

Cecal tissues were recovered, fixed for 5 h in 4% paraformaldehyde/4% sucrose, saturated in PBS/20% sucrose (overnight, 4°C), embedded in OCT medium (Sakura, Torrance, CA), snap-frozen in liquid nitrogen and stored at −80°C. Cryosections (20 µm) were air-dried, rehydrated with PBS and permeabilized with PBS/0.5% Triton X-100. Unspecific binding was blocked with PBS/10% Normal Goat Serum. The tissue sections were immunostained using rat-anti-mouse-CD18 (clone M18/2, Biolegend, (1∶300)). Goat-anti-rat-Cy3 (112-165-167, Jackson, 1∶200) was used as a secondary antibody. DNA was stained with DAPI (SIGMA Aldrich) and F-actin was stained using AlexaFluor647-conjugated phalloidin (Molecular probes). Sections were subsequently mounted with Mowiol (Calbiochem). Imaging was performed using a Zeiss Axiovert 200 m microscope equipped with 10×–100× objectives, a spinning disc confocal laser unit (Visitron) and parallel Evolve 512 EMCCD cameras (Photometrics). CD18^+^ cells were enumerated in 40× field of vision in several non-consecutive sections per mouse in a blind fashion and the average number of CD18^+^ cells were compared between experimental groups.

### Statistical analysis

The exact Mann-Whitney U test was performed using the software Graphpad Prism Version 6.0 for Windows (GraphPad Software, La Jolla California USA, www.graphpad.com). P values of less than 0.05 (two-tailed) were considered as statistically significant. * P<0.05, ** P<0.01, *** P<0.001.

Evenness indices were calculated as described [31]. In cases where none of the seven WITS tagged strains were detectable any more, no evenness index was applicable and the corresponding mice were ignored for determination of the median evenness and the comparison between evenness indices in Table 1.

It should be noted that the 1∶7 inoculum also yielded a somewhat reduced evenness by day 4 p.i. (median = 0.528). This cannot be explained by technical error when using our simple population model and may suggest that a second, so far unidentified process may affect the gut luminal *S.* Tm population. However, this effect was much smaller than the WITS-diversity loss observed with the 1∶7000 inoculum. In the present paper, we have decided to focus on the latter phenomenon.

### Estimation of the bottleneck size

We estimated the bottleneck size using the data on WITS loss after their inoculation at the lowest dilution of 1/7000. Let *w* be the number of WITS, which, in our experiments, was always *w = 7*. Further, let *m* be the number of mice in a given treatment group. Then we have a total of *mw* WITS in that treatment group. Lastly, let *x* denote the number of WITS that cannot be recovered across all mice. In the control group for example, 30 of the 70 WITS in the 10 mice were undetectable at day 4, and we thus have *w = 7*, *m = 10*, *x = 30*.

The procedure to estimate the bottleneck size is based on the simplest possible model of this process: binomial selection. We assume that during the bottleneck, *B* bacterial cells are selected. *B* is the bottleneck size, i.e. the number of bacteria that are able to traverse the bottleneck. The probability of selecting a certain WITS is determined by its dilution, *d* = 1/7000. Not selecting this WITS happens with a probability of *p*_0_ = (1−*d*)*^B^*. We can construct a log-likelihood for loosing *x* out of *mw* WITS by bottlenecking: ℓ = *x*ln*p*_0_+(*mw*−*x*)ln(1−*p*_0_). Maximizing this log-likelihood yields the maximum likelihood estimates for the bottleneck size listed in Table 2. To calculate confidence intervals for the estimates we used the profile likelihood [78]. If all or no WITS are lost in a treatment group, only upper or lower bounds of the bottleneck size can be calculated, respectively.

### Ethical statement

All animal experiments were reviewed and approved by the Kantonales Veterinäramt, Zürich (license 223/2010) and are subject to the Swiss animal protection law (TschG).

## Supporting Information

S1 Figure **Schematic overview of the experimental setup to investigate WITS diversity loss in mouse models for oral** ***S.*** **Tm infections.** (A) Mice were infected with a mixture of 7 individually tagged *S.* Tm strains (WITS = wild type isogenic tagged strains) in equal proportions. *S.* Tm population composition in feces and cecal contents of these mice were monitored by collecting samples, selectively growing the tagged strains based on their antibiotic resistance marker, isolating gDNA of the tagged population and quantifying each of the tagged strains by rtqPCR using tag-specific primers. (B) WITS proportions after the infection experiment indicate whether tagged strains are randomly lost. WITS diversity was measured by an evenness index, ranging from 1 (totally even) to 0 (totally uneven). (C) Dilution of the tagged strains with the untagged, isogenic background strain enhances the sensitivity of WITS diversity loss detection, resulting in a more variable WITS composition with a random dominant tagged strain.
(TIF)

S2 Figure **Enrichment culturing does not bias WITS analysis.** To test whether culturing of cecal/fecal samples to enrich for the WITS-tagged subpopulation biases WITS composition analysis, we performed serial dilution of the tagged strains, inoculated overnight cultures and analyzed WITS population evenness after incubation. The horizontal grey line depicts the detection limit. Blue numbers indicate the evenness index/the median of the evenness indices of each replicate. Starting at inoculation sizes as low as 20 bacteria, WITS evenness decreases due to sampling effects.
(TIF)

S3 Figure **Selective sweep is not sufficient to account for the WITS diversity loss shown in** **Fig. 1** **and** **Fig. 2** **.** To exclude that fitness-enhancing mutants might confound our analysis, we performed competitive infection experiments with clones re-isolated at day 4 p.i. Selective sweep by such mutants with higher fitness than wild type *S.* Tm may potentially result in the loss of WITS-diversity shown in Fig. 1. Earlier work has shown that *S.* Tm mutants with increased fitness can indeed be selected during within-host evolution [39]. Occurrence of beneficial mutations is a random process attributable to the standard mutation rate (approximately 0.3–1.5×10^−9^ mutations per base pair per generation for *S.* Tm LT2 [79]) that may result in particular fit clones which can outcompete (exclude) the rest of the population. Beneficial mutations could have been selected in the untagged populations of *S.* Tm or in the WITS-tagged subpopulations. To test this hypothesis we isolated the most frequent clones from stool at day 4 p.i. Such clones should be representative of the surviving population and might therefore harbor beneficial mutations. We isolated representatives of the untagged population (“untagged”) and of the tagged population (“tagged”). To test their relative fitness, streptomycin pretreated C57BL/6 mice were infected with a 1∶1 mixture of the re-isolate and of the isogenic original *S.* Tm strain (5×10^7^ cfu total, by gavage) and we analyzed pathogen loads in the cecal content at day 4 p.i. by plating. We compared the fitness of the isolate to a “control” strain, i.e. a naïve WITS strain as used for inoculum preparation in our infection experiments. Five out of six re-isolates had competitive index (C.I.) values of 1, indicating that they featured equivalent fitness than the wild type strain (and a naïve “control” WITS strain). Thus, selective sweep cannot explain the loss of WITS-diversity in the experiment shown in Fig. 1 and Fig. 2. In conclusion, these data support that a bottleneck in the pathogen population might reduce the WITS diversity in the gut lumen of *S.* Tm^WT^ infected mice. C.I. = ratio of the isolate to the background strains in stool at day 4 p.i. divided by the ratio of both strains in the inoculum. The grey line represents a C.I. of 1 indicating that the naïve background strain and the isolate are equally fit.
(TIF)

S4 Figure **Supplementary information on the experiment depicted in** **Fig. 2** **.** *S.* Tm loads of the untagged and tagged strains were monitored in the mLN (A), spleen (B) and liver (C) of mice infected with the mixture of the WITS strains in an equal ratio, diluted by untagged *S.* Tm^WT^ to a final dilution of each of the tagged strains of “1∶7000”. This pattern of colonization is in line with previous work. There are two interesting observations that should be pointed out. First, the pathogen loads of livers and spleens differed significantly from animal-to-animal at day 1 p.i. In some cases, the loads were even higher than at days 2 p.i. This would be in line with earlier observations indicating that the systemic spread may occur in two separate waves. During the first hours of an orogastric infection work in *S.* Tm and *Yersinia* infection models identified initial (and quite random) “waves” of bacteria leaving the gut, but failing to establish stable populations at systemic sites [3], [80]. A second wave of bacteria is thought to colonize these sites starting by day 2 p.i. In our case, this would correspond to the steady increase in spleen and liver organ loads by about 1.5 log_10_ per day from day 2 to day 4 p.i. Second, it is interesting to note that no animal harbored any WITS in the mLN, spleen or the liver. This is in line with our previous work [6], [29] and indicates that these sites are seeded by much fewer than 1000 bacteria (estimated 385 bacteria) and that the few “initial colonizers” have been replicating on site to fill the respective niche. Grey lines depict the detection limit for plating.
(TIF)

S5 Figure **Supplementary information on experiments depicted in** **Fig. 6** **. Anti-Gr-1 neutralization efficacy on inflammatory myeloids in the cecal mucosa.** (A–B) PBS or anti-Gr-1-treated 129 Sv/Ev mice were infected with *S.* Tm^WT^ and sacrificed at the indicated time points. The number of granulocytes, as well as inflammatory monocytes was determined in the cecal mucosa during infection (S1 Text). (A) Gating strategy to identify the denoted myeloid subsets. Frequency on plots represent percentage of either CD45^+^ CD3^−^ CD11b^+^ live cells (upper panel) or CD45^+^ MHCII^−^ live cells (lower panel). (B) Number of Siglec-F^+^ Ly6G^−^ eosinophils, Siglec-F^−^ Ly6G^+^ neutrophils and CD11b^+^ Ly6C^hi^ monocytes are plotted at day 1 and 2 post infection. Of note, quantification in B are complementary data to Fig. 6C, D and E from the same ceca.
(TIF)

S6 Figure **Pilot experiments to specifically deplete granulocytes by injection of anti-G-CSF and anti-Ly6G antibodies confirm that WITS diversity loss is granulocyte-dependent.** (A) Schematic overview on the experimental setup to specifically deplete granulocytes (S1 Text). (B) Flow cytometry of blood derived from the G-CSF/Ly6G-depleted 129 Sv/Ev mice and the control group (treated with isotype control antibodies) confirm specific depletion of granulocytes, while monocytes are still present. (C) WITS analysis of the fecal (day 1 p.i.) and cecal (day 3 p.i.) *S.* Tm population in G-CSF/Ly6G depleted mice compared to the control group. Grey lines indicate the detection limit for plating (10 cfu/g) and blue numbers indicated the median of the evenness indices of single mice.
(TIF)

S7 Figure **Deficiency in host-derived NO does not alleviate WITS diversity loss.** *Nos2*-deficient mice and their heterozygous littermates were infected with a cocktail of the 7 WITS strains in a 1∶7000 dilution. For each mouse (mouse identifiers on x-axis) the cfu/g fecal (day 1 p.i.) and cecal content (day 3 p.i.) (A) and the cfu per mLN, liver and spleen (B) of the total population and the individual WITS strains are depicted. Grey lines indicate the detection limit for plating (10 cfu/g for feces, cecum and mLN, 20 cfu/organ for spleen, 60 cfu/organ for liver) and blue numbers indicate the median of the evenness indices of single mice. (C) Cecal pathological scoring at day 3 p.i. indicated intestinal inflammation. (D) Analysis of the inocula used for the experiments depicted in this figure and in Fig. S8 verified a 1∶7000 ratio of each of the tagged strains.
(TIF)

S8 Figure **ROS formation might partially explain the WITS diversity loss in the lumen of the inflamed gut.** *Cybb*-deficient mice and a heterozygous control group were inoculated with a mixture of the 7 WITS strains in a 1∶7000 dilution (see analysis of inoculum in panel D of suppl. Fig. S7). The composition of the *S.* Tm population in fecal (day 1 p.i.) and cecal content (day 3 p.i.) (A), as well as in mLN, spleen and liver (B) is shown for each animal (mouse identifiers are plotted on the x-axis). Please note that *S.* Tm colonization at systemic sites is one to two orders of magnitude higher in the *Cybb−/−* mice compared to the heterozygous control group. Grey lines indicate the detection limit for plating (10 cfu/g for feces, cecum and mLN, 20 cfu/organ for spleen, 60 cfu/organ for liver) and blue numbers indicate the median of the evenness indices of single mice. (C) Analysis of cecal sections at day 3 p.i. by pathoscoring revealed severe intestinal inflammation.
(TIF)

S1 Table **Strains used in this study.**
(DOCX)

S2 Table **Bottleneck estimates in iNOS- and Cybb-deficient animals.**
(DOCX)

S1 Text **Supplementary materials and methods.**
(DOCX)
