# Correction: Granulocytes Impose a Tight Bottleneck upon the Gut Luminal Pathogen Population during *Salmonella* Typhimurium Colitis

**DOI:** 10.1371/journal.ppat.1005047

**Published:** 2015-07-17

**Authors:** Lisa Maier, Médéric Diard, Mikael E. Sellin, Elsa-Sarah Chouffane, Kerstin Trautwein-Weidner, Balamurugan Periaswamy, Emma Slack, Tamas Dolowschiak, Bärbel Stecher, Claude Loverdo, Roland R. Regoes, Wolf-Dietrich Hardt

There are errors in the Author Contributions. The correct contributions are: Conceived and designed the experiments: LM MD MES BP ES BS WDH. Performed the experiments: LM MD MES ESC TD KTW. Analyzed the data: LM MD MES ESC BP ES BS TD KTW. Contributed reagents/materials/analysis tools: KTW TD. Wrote the paper: LM WDH. Computational analysis: CL RRR.
