# Supplementary figures and images for: Granulocytes Impose a Tight Bottleneck upon the Gut Luminal Pathogen Population during Salmonella Typhimurium Colitis
Source: PLoS Pathog. 2014 Dec 18;10(12):e1004557. doi: 10.1371/journal.ppat.1004557 (PMC4270771; doi:10.1371/journal.ppat.1004557)

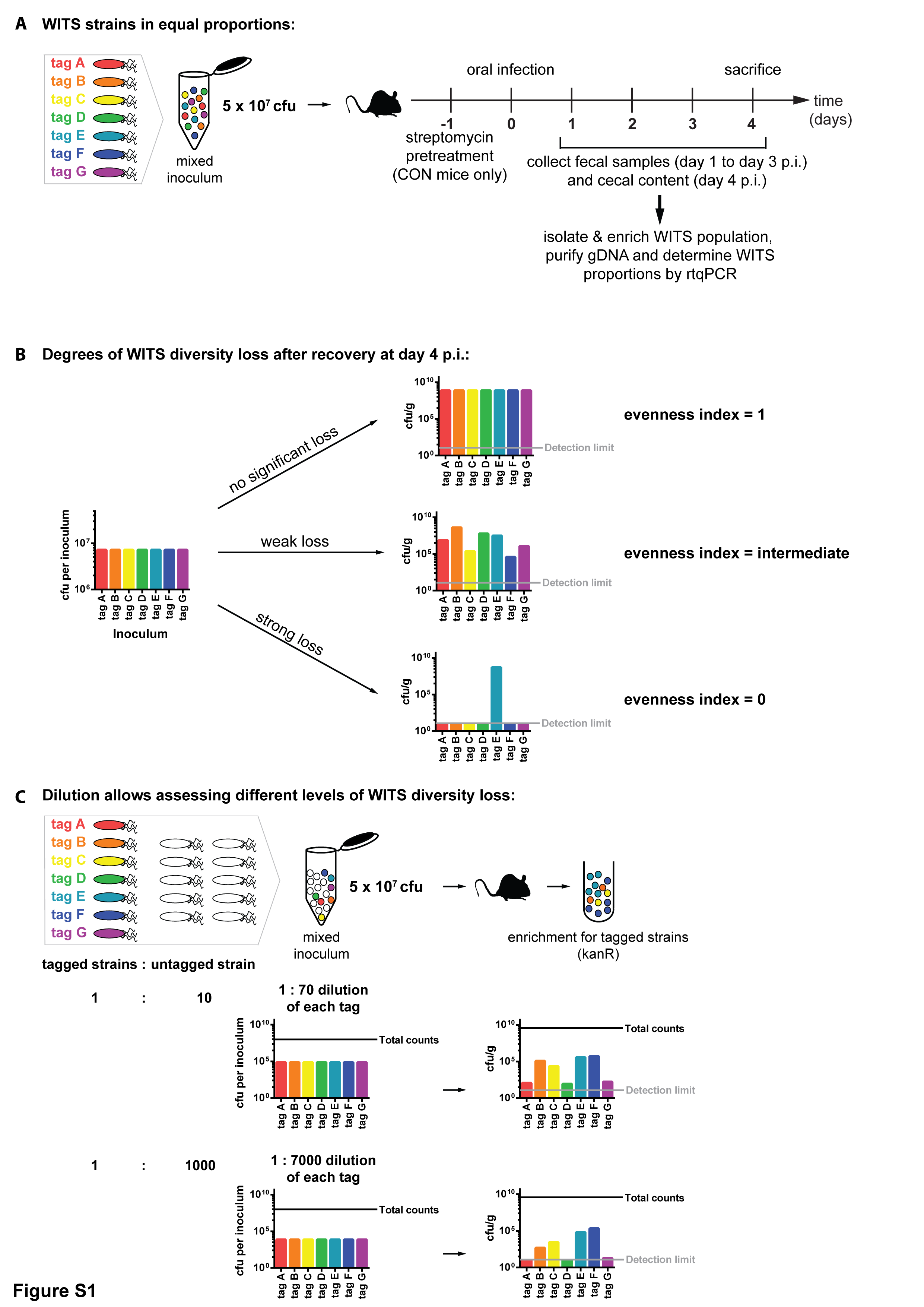

Supplement: S1 Figure — Schematic overview of the experimental setup to investigate WITS diversity loss in mouse models for oral S. Tm infections. (A) Mice were infected with a mixture of 7 individually tagged S. Tm strains (WITS = wild type isogenic tagged strains) in equal proportions. S. Tm population composition in feces and cecal contents of these mice were monitored by collecting samples, selectively growing the tagged strains based on their antibiotic resistance marker, isolating gDNA of the tagged population and quantifying each of the tagged strains by rtqPCR using tag-specific primers. (B) WITS proportions after the infection experiment indicate whether tagged strains are randomly lost. WITS diversity was measured by an evenness index, ranging from 1 (totally even) to 0 (totally uneven). (C) Dilution of the tagged strains with the untagged, isogenic background strain enhances the sensitivity of WITS diversity loss detection, resulting in a more variable WITS composition with a random dominant tagged strain. (TIF) [file ppat.1004557.s001.tif]

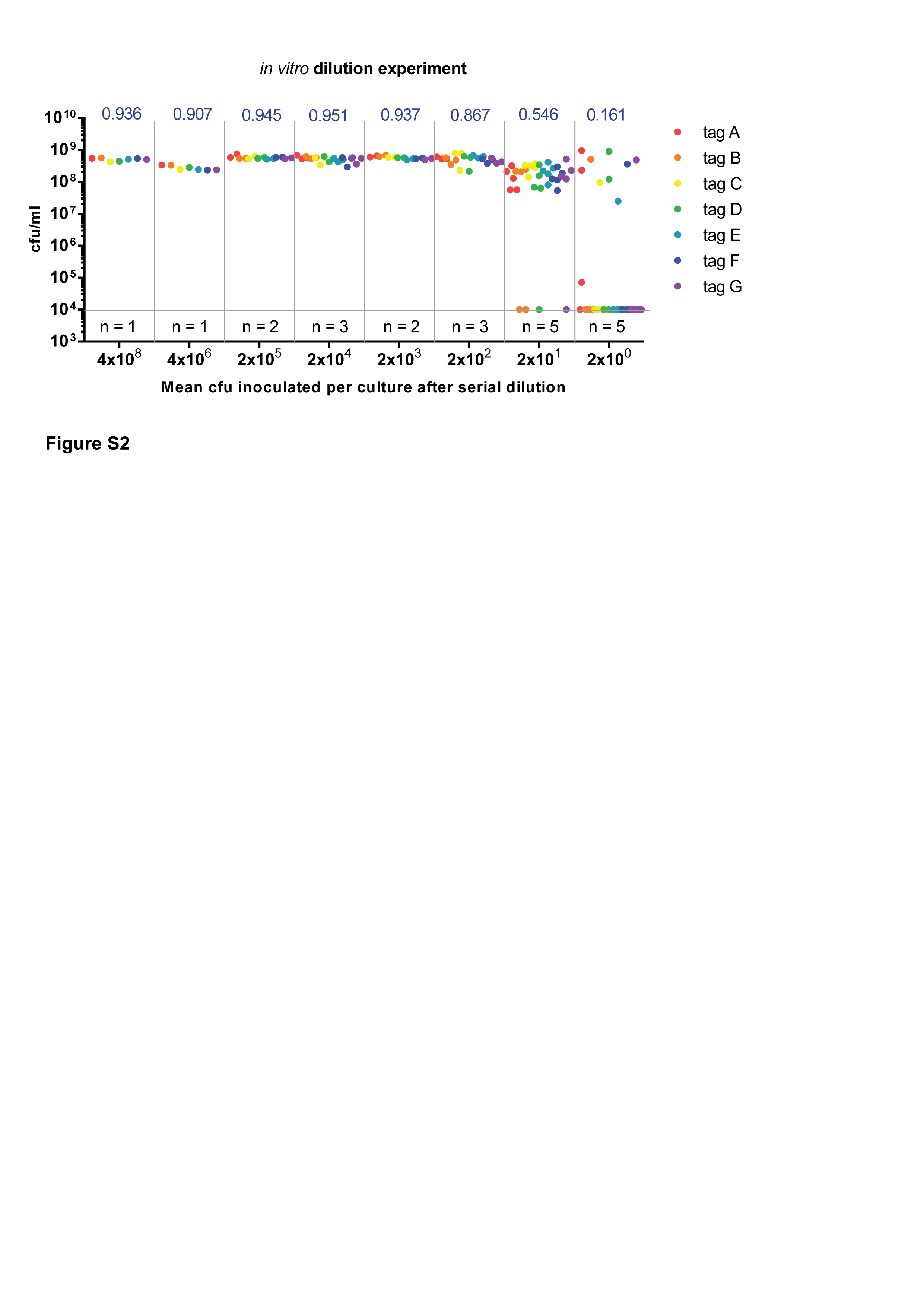

Supplement: S2 Figure — Enrichment culturing does not bias WITS analysis. To test whether culturing of cecal/fecal samples to enrich for the WITS-tagged subpopulation biases WITS composition analysis, we performed serial dilution of the tagged strains, inoculated overnight cultures and analyzed WITS population evenness after incubation. The horizontal grey line depicts the detection limit. Blue numbers indicate the evenness index/the median of the evenness indices of each replicate. Starting at inoculation sizes as low as 20 bacteria, WITS evenness decreases due to sampling effects. (TIF) [file ppat.1004557.s002.tif]

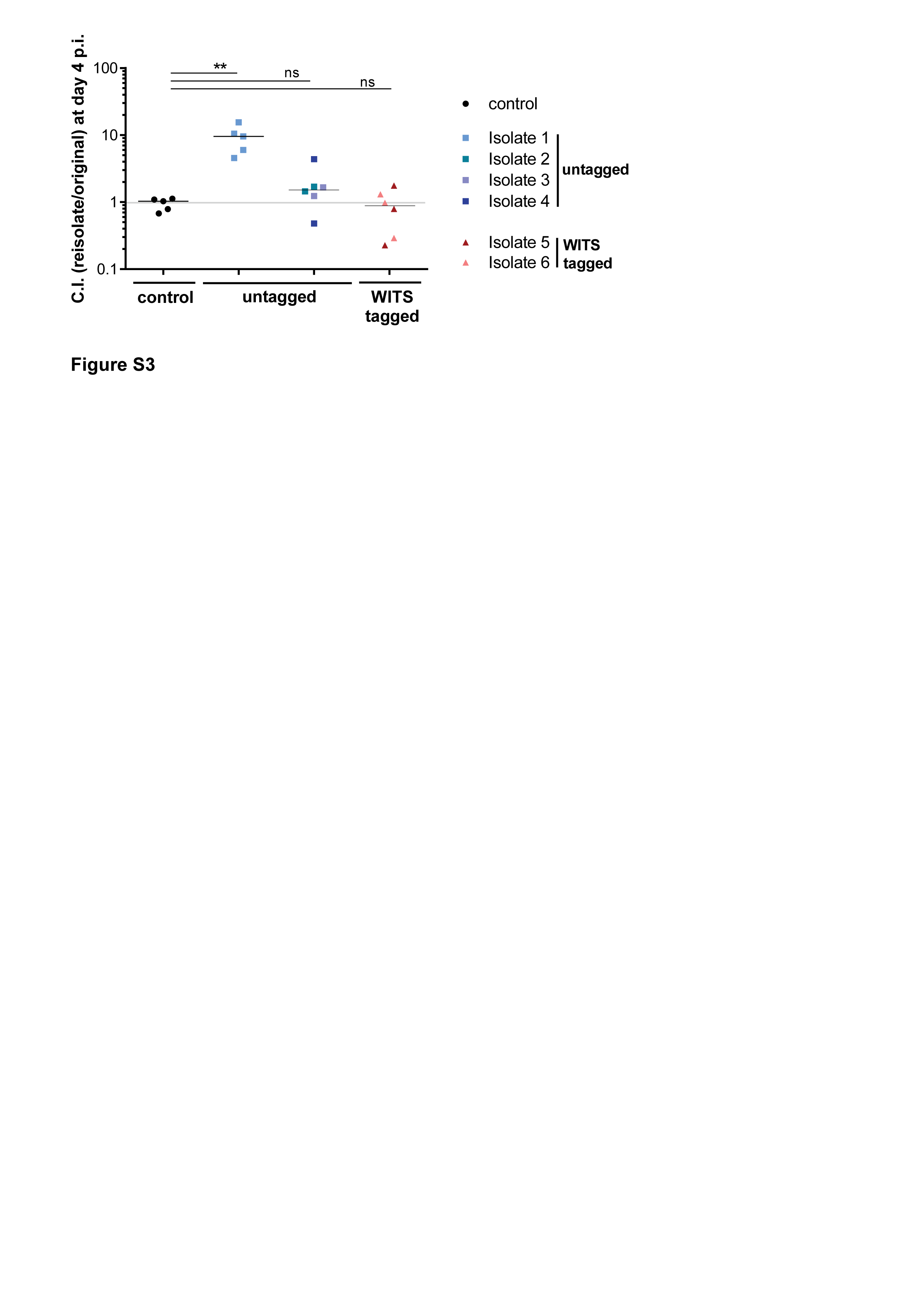

Supplement: S3 Figure — Selective sweep is not sufficient to account for the WITS diversity loss shown in Fig. 1 and Fig. 2 . To exclude that fitness-enhancing mutants might confound our analysis, we performed competitive infection experiments with clones re-isolated at day 4 p.i. Selective sweep by such mutants with higher fitness than wild type S. Tm may potentially result in the loss of WITS-diversity shown in Fig. 1. Earlier work has shown that S. Tm mutants with increased fitness can indeed be selected during within-host evolution [39]. Occurrence of beneficial mutations is a random process attributable to the standard mutation rate (approximately 0.3–1.5×10^−9^ mutations per base pair per generation for S. Tm LT2 [79]) that may result in particular fit clones which can outcompete (exclude) the rest of the population. Beneficial mutations could have been selected in the untagged populations of S. Tm or in the WITS-tagged subpopulations. To test this hypothesis we isolated the most frequent clones from stool at day 4 p.i. Such clones should be representative of the surviving population and might therefore harbor beneficial mutations. We isolated representatives of the untagged population (“untagged”) and of the tagged population (“tagged”). To test their relative fitness, streptomycin pretreated C57BL/6 mice were infected with a 1∶1 mixture of the re-isolate and of the isogenic original S. Tm strain (5×10^7^ cfu total, by gavage) and we analyzed pathogen loads in the cecal content at day 4 p.i. by plating. We compared the fitness of the isolate to a “control” strain, i.e. a naïve WITS strain as used for inoculum preparation in our infection experiments. Five out of six re-isolates had competitive index (C.I.) values of 1, indicating that they featured equivalent fitness than the wild type strain (and a naïve “control” WITS strain). Thus, selective sweep cannot explain the loss of WITS-diversity in the experiment shown in Fig. 1 and Fig. 2. In conclusion, these data support that a bottlenec [file ppat.1004557.s003.tif]

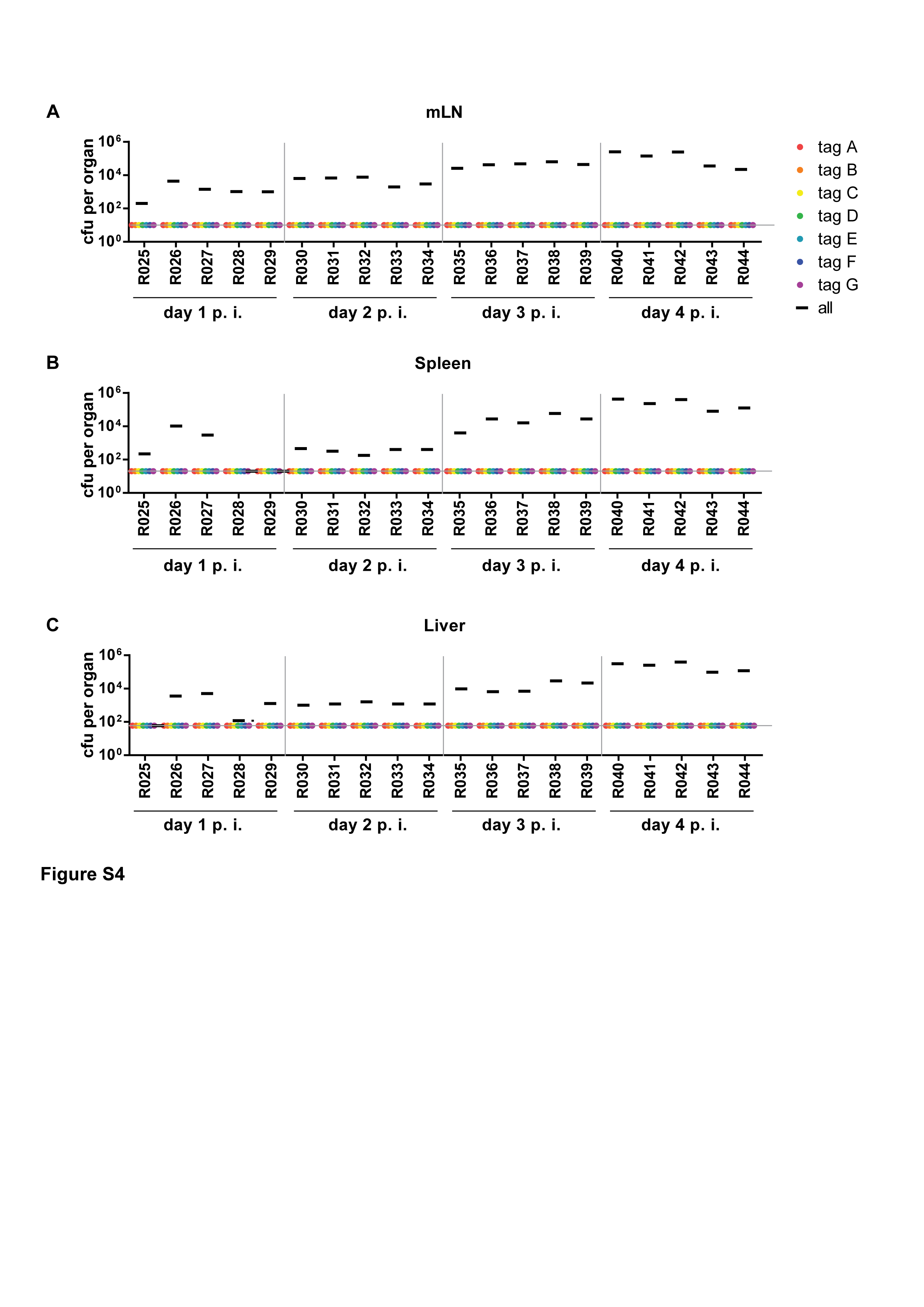

Supplement: S4 Figure — Supplementary information on the experiment depicted in Fig. 2 . S. Tm loads of the untagged and tagged strains were monitored in the mLN (A), spleen (B) and liver (C) of mice infected with the mixture of the WITS strains in an equal ratio, diluted by untagged S. TmWT to a final dilution of each of the tagged strains of “1∶7000”. This pattern of colonization is in line with previous work. There are two interesting observations that should be pointed out. First, the pathogen loads of livers and spleens differed significantly from animal-to-animal at day 1 p.i. In some cases, the loads were even higher than at days 2 p.i. This would be in line with earlier observations indicating that the systemic spread may occur in two separate waves. During the first hours of an orogastric infection work in S. Tm and Yersinia infection models identified initial (and quite random) “waves” of bacteria leaving the gut, but failing to establish stable populations at systemic sites [3], [80]. A second wave of bacteria is thought to colonize these sites starting by day 2 p.i. In our case, this would correspond to the steady increase in spleen and liver organ loads by about 1.5 log10 per day from day 2 to day 4 p.i. Second, it is interesting to note that no animal harbored any WITS in the mLN, spleen or the liver. This is in line with our previous work [6], [29] and indicates that these sites are seeded by much fewer than 1000 bacteria (estimated 385 bacteria) and that the few “initial colonizers” have been replicating on site to fill the respective niche. Grey lines depict the detection limit for plating. (TIF) [file ppat.1004557.s004.tif]

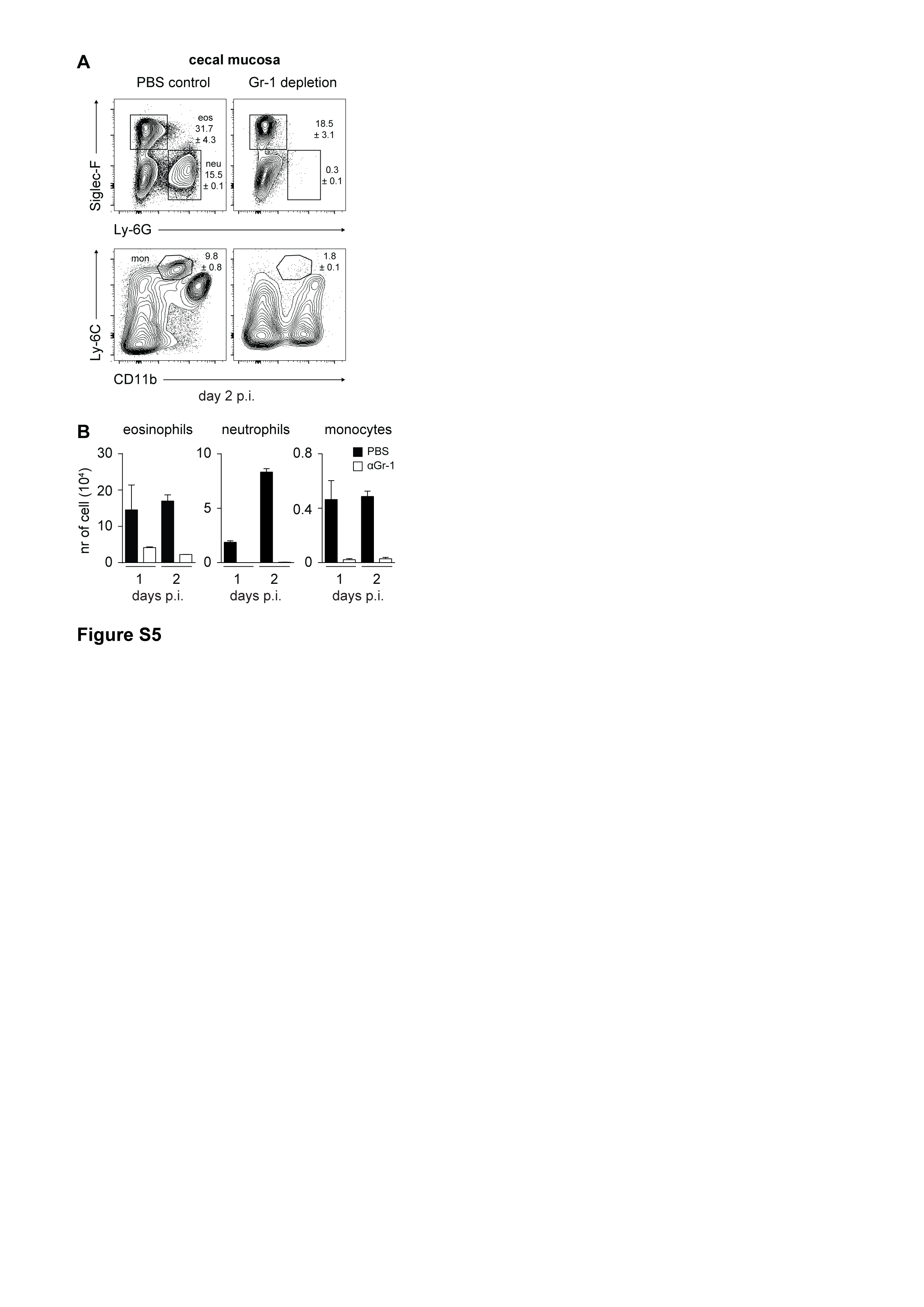

Supplement: S5 Figure — Supplementary information on experiments depicted in Fig. 6 . Anti-Gr-1 neutralization efficacy on inflammatory myeloids in the cecal mucosa. (A–B) PBS or anti-Gr-1-treated 129 Sv/Ev mice were infected with S. TmWT and sacrificed at the indicated time points. The number of granulocytes, as well as inflammatory monocytes was determined in the cecal mucosa during infection (S1 Text). (A) Gating strategy to identify the denoted myeloid subsets. Frequency on plots represent percentage of either CD45+ CD3− CD11b+ live cells (upper panel) or CD45+ MHCII− live cells (lower panel). (B) Number of Siglec-F+ Ly6G− eosinophils, Siglec-F− Ly6G+ neutrophils and CD11b+ Ly6Chi monocytes are plotted at day 1 and 2 post infection. Of note, quantification in B are complementary data to Fig. 6C, D and E from the same ceca. (TIF) [file ppat.1004557.s005.tif]

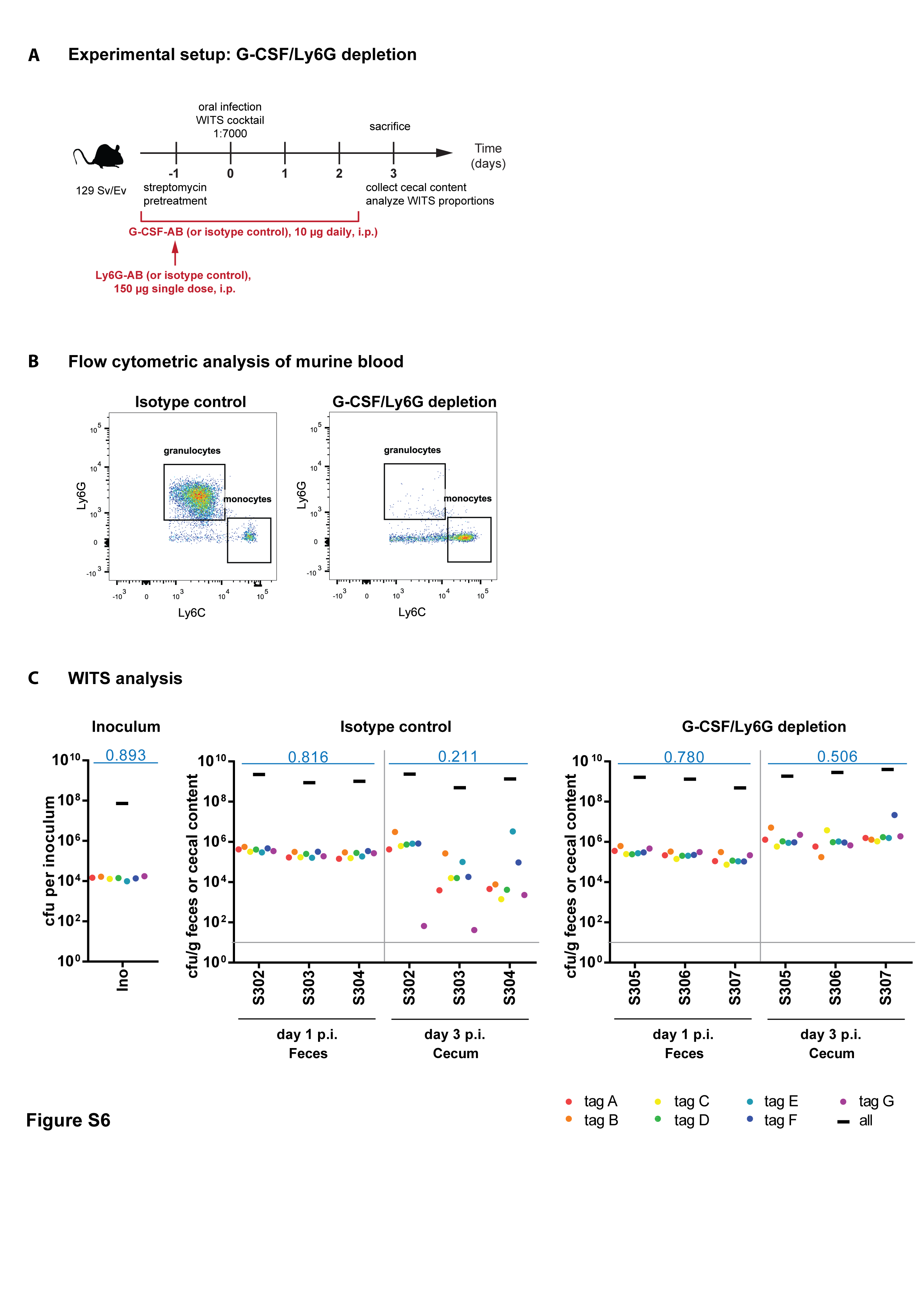

Supplement: S6 Figure — Pilot experiments to specifically deplete granulocytes by injection of anti-G-CSF and anti-Ly6G antibodies confirm that WITS diversity loss is granulocyte-dependent. (A) Schematic overview on the experimental setup to specifically deplete granulocytes (S1 Text). (B) Flow cytometry of blood derived from the G-CSF/Ly6G-depleted 129 Sv/Ev mice and the control group (treated with isotype control antibodies) confirm specific depletion of granulocytes, while monocytes are still present. (C) WITS analysis of the fecal (day 1 p.i.) and cecal (day 3 p.i.) S. Tm population in G-CSF/Ly6G depleted mice compared to the control group. Grey lines indicate the detection limit for plating (10 cfu/g) and blue numbers indicated the median of the evenness indices of single mice. (TIF) [file ppat.1004557.s006.tif]

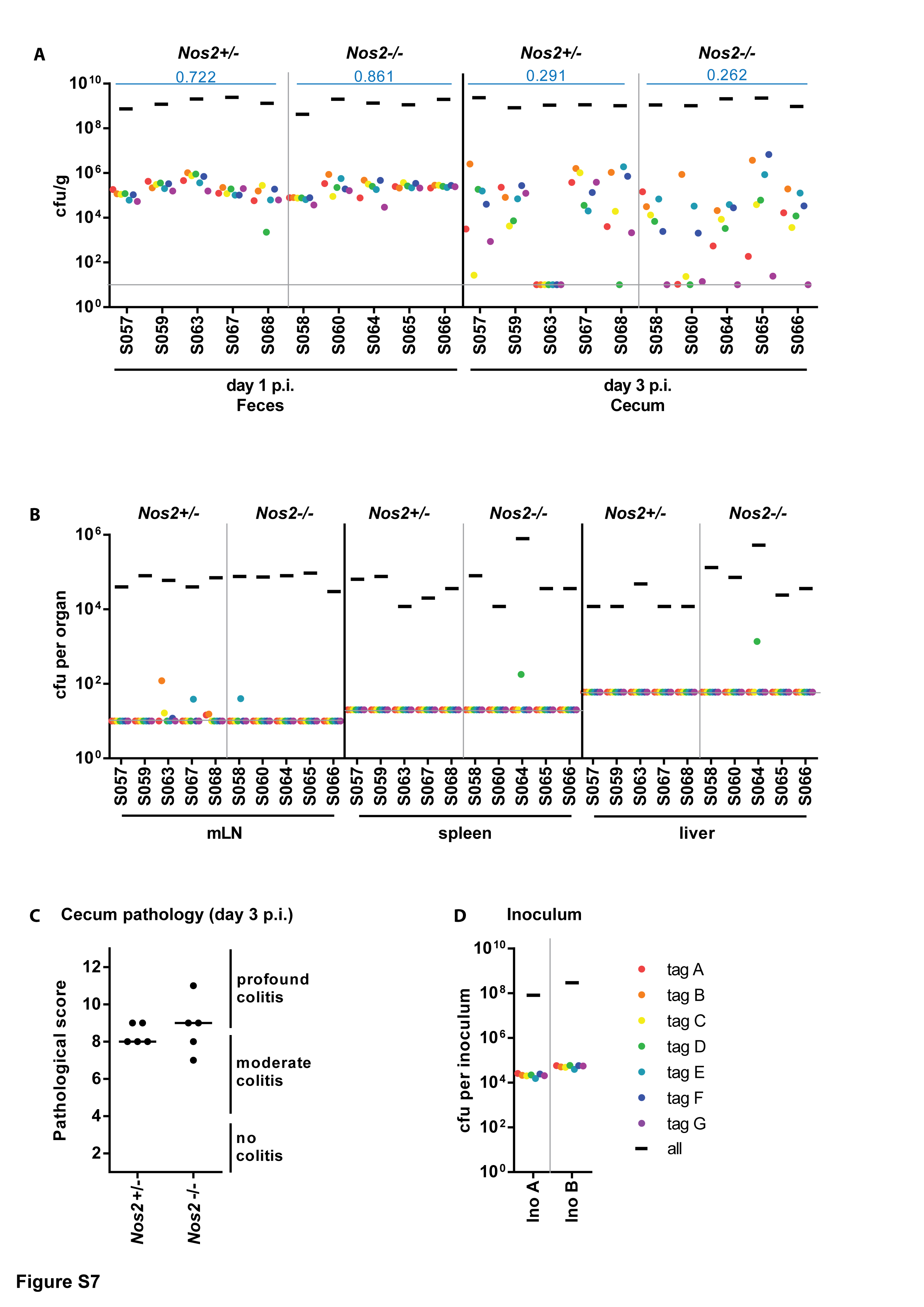

Supplement: S7 Figure — Deficiency in host-derived NO does not alleviate WITS diversity loss. Nos2-deficient mice and their heterozygous littermates were infected with a cocktail of the 7 WITS strains in a 1∶7000 dilution. For each mouse (mouse identifiers on x-axis) the cfu/g fecal (day 1 p.i.) and cecal content (day 3 p.i.) (A) and the cfu per mLN, liver and spleen (B) of the total population and the individual WITS strains are depicted. Grey lines indicate the detection limit for plating (10 cfu/g for feces, cecum and mLN, 20 cfu/organ for spleen, 60 cfu/organ for liver) and blue numbers indicate the median of the evenness indices of single mice. (C) Cecal pathological scoring at day 3 p.i. indicated intestinal inflammation. (D) Analysis of the inocula used for the experiments depicted in this figure and in Fig. S8 verified a 1∶7000 ratio of each of the tagged strains. (TIF) [file ppat.1004557.s007.tif]

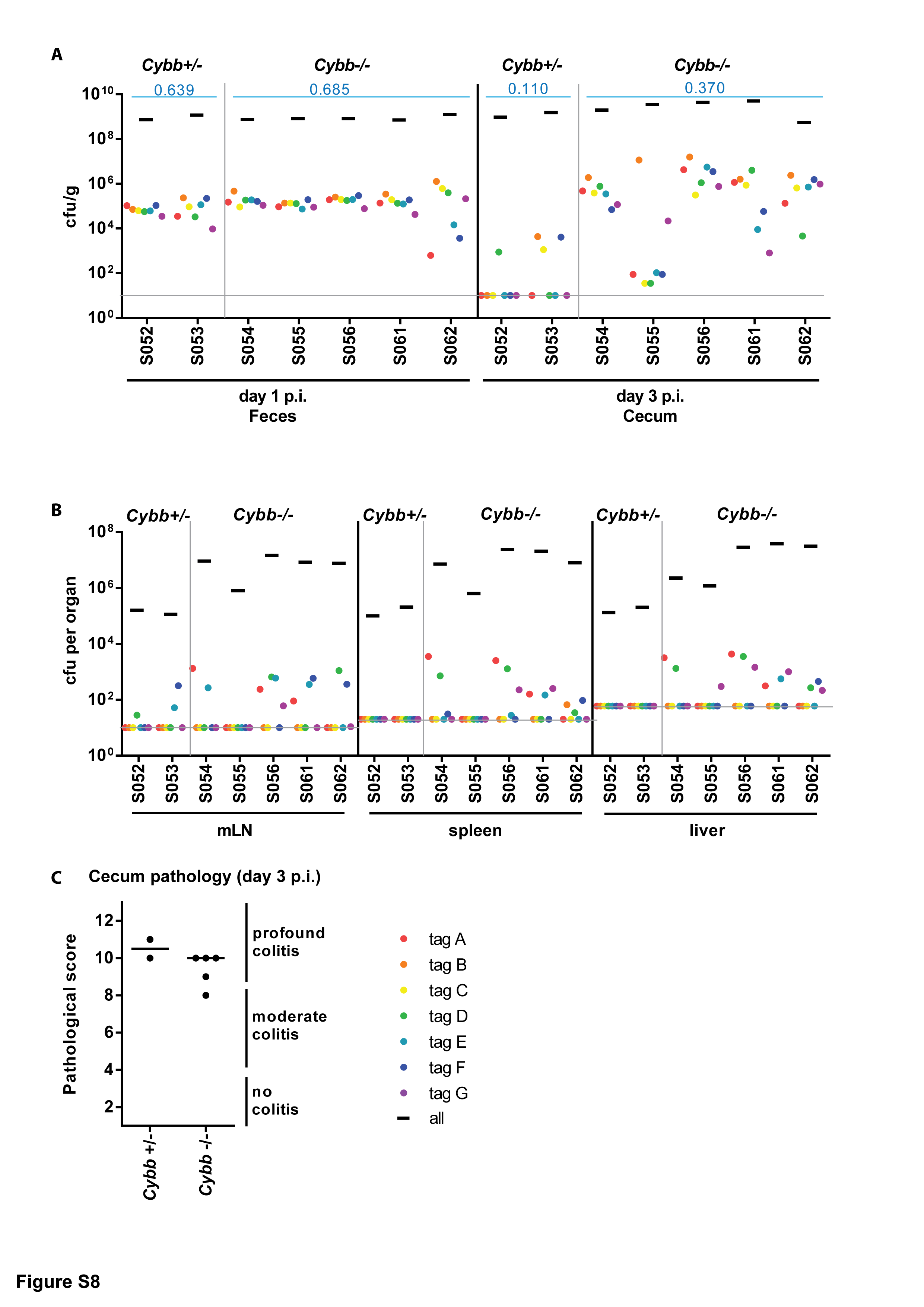

Supplement: S8 Figure — ROS formation might partially explain the WITS diversity loss in the lumen of the inflamed gut. Cybb-deficient mice and a heterozygous control group were inoculated with a mixture of the 7 WITS strains in a 1∶7000 dilution (see analysis of inoculum in panel D of suppl. Fig. S7). The composition of the S. Tm population in fecal (day 1 p.i.) and cecal content (day 3 p.i.) (A), as well as in mLN, spleen and liver (B) is shown for each animal (mouse identifiers are plotted on the x-axis). Please note that S. Tm colonization at systemic sites is one to two orders of magnitude higher in the Cybb−/− mice compared to the heterozygous control group. Grey lines indicate the detection limit for plating (10 cfu/g for feces, cecum and mLN, 20 cfu/organ for spleen, 60 cfu/organ for liver) and blue numbers indicate the median of the evenness indices of single mice. (C) Analysis of cecal sections at day 3 p.i. by pathoscoring revealed severe intestinal inflammation. (TIF) [file ppat.1004557.s008.tif]
