# Supplementary material for: Granulocytes Impose a Tight Bottleneck upon the Gut Luminal Pathogen Population during Salmonella Typhimurium Colitis
Source: PLoS Pathog. 2014 Dec 18;10(12):e1004557. doi: 10.1371/journal.ppat.1004557 (PMC4270771; doi:10.1371/journal.ppat.1004557)
Supplement: S1 Table — Strains used in this study. (DOCX) [file ppat.1004557.s009.docx]

**Supplementary Table S1: Strains used in this study**

| Strain |  | Genotype | Reference |
| --- | --- | --- | --- |
| *S.* Tm^WT^ | SB300 | Wild-type *S*. Typhimurium SL1344, *hisG* | [[74](#_ENREF_74)] |
| WITS^WT^ | M2608-2613, M2615 | Wild-type *S*. Typhimurium SL1344, *hisG*, WITS-tagged | [[5](#_ENREF_5)] |
| *S.* Tm^SPI-1 & SPI-2^ | M2702 | *S*. Tm Δ*invG*, Δ*ssaV* | [[17](#_ENREF_17)] |
| WITS^SPI-1 & SPI-2^ | M3078-M3084 | *S*. Tm Δ*invG*, Δ*ssaV*, WITS-tagged | This study |
| *S.* Tm^SPI-1^ | SB161 | SB161 (*ΔinvG*) | [[34](#_ENREF_34)] |
| WITS^SPI-1^ | M2616-M2621, M2623 | SB161 (*ΔinvG*) , WITS-tagged | [[6](#_ENREF_6)] |
| *S.* Tm^SPI-2^ | M1516 | M1516 (*ssaV::cat*) | [[35](#_ENREF_35)] |
| WITS^SPI-2^ | M2731-M2737 | M1516 (*ssaV::cat*), WITS-tagged | This study |
