# Supplementary material for: Granulocytes Impose a Tight Bottleneck upon the Gut Luminal Pathogen Population during Salmonella Typhimurium Colitis
Source: PLoS Pathog. 2014 Dec 18;10(12):e1004557. doi: 10.1371/journal.ppat.1004557 (PMC4270771; doi:10.1371/journal.ppat.1004557)
Supplement: S2 Table — Bottleneck estimates in iNOS- and Cybb-deficient animals. (DOCX) [file ppat.1004557.s010.docx]

**Supplementary Table S2: Bottleneck estimates in iNOS- and Cybb-deficient animals**

|  | Bottleneck estimate | Confidence interval  Lower bound | Confidence interval  Higher bound |
| --- | --- | --- | --- |
| Cybb -/-, day 3 p. i., Cecum | NA | 20509 | NA |
| iNOS +/-, day 3 p. i., Cecum | 10331 | 6703 | 15344 |
| iNOS -/-, day 3 p. i., Cecum | 15182 | 9821 | 23083 |
