# Supplementary material for: Granulocytes Impose a Tight Bottleneck upon the Gut Luminal Pathogen Population during Salmonella Typhimurium Colitis
Source: PLoS Pathog. 2014 Dec 18;10(12):e1004557. doi: 10.1371/journal.ppat.1004557 (PMC4270771; doi:10.1371/journal.ppat.1004557)
Supplement: S1 Text — Supplementary materials and methods. (DOCX) [file ppat.1004557.s011.docx]

**Supplementary Materials and Methods:**

***Lamina propria cell staining and flow cytometric analysis.*** Intestinal tissue was opened longitudinally, placed into ice cold PBS and shortly vortexed in order to release remaining luminal content. Ceca were cut into fine pieces and placed in consecutive rounds into PBS containing 5 mM EDTA, 15 mM HEPES and 10 % FCS for 20 min at 37°C while mildly shaking at 160 rpm. Gut samples were then washed in RPMI supplemented with 30 % FCS and digested in RPMI supplemented with Collagenase VIII (1 mg/ml, Sigma) and DNase I (0.2 mg/ml, Roche). Cells were passed through a 70 µm cell strainer and washed with RPMI. Isolated cells in RPMI were layered on a NycoPrep 1.077^TM^ matrix (Progen) and centrifuged for 30 min at 400 g. Cells at the interface were collected and rinsed in RPMI. Single cell suspensions were stained in ice-cold PBS supplemented with 10 % FCS and 0.02% sodium azide. Antibodies were either from Biolegend, as CD45 (clone 30-F11), CD3 (17A2), CD11b (M1/70) and MHCII (M5/114.15.2), or from BD Biosciences, as Siglec-F (E50-2440), Ly6G (1A8) and Ly6C (AL-21). For live/dead measurement, SYTOX stain (Invitrogen) was used. Data were acquired on a LSRII (BD Biosciences) and analyzed with FlowJo software (TreeStar).

***G-CSF/Ly6G depletion.*** Granulocytes were depleted by a daily intraperitoneal application of anti-G-CSF (clone 67604, R&D Systems (Abingdon, UK), 10 µg per mouse per day) starting one day prior to infection in combination with a single dose of anti-Ly6G (clone 1A8, BioXCell, 150 µg per mouse) at day -1 as described previously [[48](#_ENREF_48),[49](#_ENREF_49)]. As isotype control antibodies, we applied rat lgG2a (anti trinitrophenol, clone 2A3, BioXCell) for anti-Ly6G and rat IgG1 (anti horseradish peroxidase, clone HRPN, BioXCell) for anti-GCSF.
